# Microtubule disruption reduces metastasis more effectively than primary tumor growth

**DOI:** 10.1186/s13058-022-01506-2

**Published:** 2022-02-14

**Authors:** Keyata N. Thompson, Julia A. Ju, Eleanor C. Ory, Stephen J. P. Pratt, Rachel M. Lee, Trevor J. Mathias, Katarina T. Chang, Cornell J. Lee, Olga G. Goloubeva, Patrick C. Bailey, Kristi R. Chakrabarti, Christopher M. Jewell, Michele I. Vitolo, Stuart S. Martin

**Affiliations:** 1 Marlene and Stewart Greenebaum NCI Comprehensive Cancer Center, University of Maryland School of Medicine, 655 W. Baltimore Street, Bressler Research Building, Rm 10-029, Baltimore, MD 21201 USA; 2 Department of Biochemistry and Molecular Biology, University of Maryland, Baltimore, MD USA; 3 Program in Molecular Medicine, University of Maryland Graduate Program in Life Sciences, Baltimore, USA; 4 Department of Epidemiology and Public Health, University of Maryland, Baltimore, MD USA; 5 Fischell Department of Bioengineering, University of Maryland, College Park, MD USA; 6 Department of Physiology, University of Maryland School of Medicine, Baltimore, MD USA; 7 United States Department of Veterans Affairs, VA Maryland Health Care System, Baltimore, MD USA

**Keywords:** Vinorelbine, Metastasis, Breast cancer, Microtentacles, Reattachment, MDA-MB-231, Circulating tumor cells

## Abstract

Clinical cancer imaging focuses on tumor growth rather than metastatic phenotypes. The microtubule-depolymerizing drug, Vinorelbine, reduced the metastatic phenotypes of microtentacles, reattachment and tumor cell clustering more than tumor cell viability. Treating mice with Vinorelbine for only 24 h had no significant effect on primary tumor survival, but median metastatic tumor survival was extended from 8 to 30 weeks. Microtentacle inhibition by Vinorelbine was also detectable within 1 h, using tumor cells isolated from blood samples. As few as 11 tumor cells were sufficient to yield 90% power to detect this 1 h Vinorelbine drug response, demonstrating feasibility with the small number of tumor cells available from patient biopsies. This study establishes a proof-of-concept that targeted microtubule disruption can selectively inhibit metastasis and reveals that existing FDA-approved therapies could have anti-metastatic actions that are currently overlooked when focusing exclusively on tumor growth.

**Supplementary Information:**

The online version contains supplementary material available at 10.1186/s13058-022-01506-2.

## Introduction

Metastasis is responsible for approximately 90% of breast cancer patient deaths, rather than growth of the primary tumor [1]. However, both breast cancer detection and monitoring currently rely on imaging modalities (mammogram, MRI, PET-CT) that require a tumor foci of more than 10 million tumor cells to reach the threshold of clinical detection [2]. As an inevitable result of this detection limit, patient management and drug development for breast cancer are predominantly focused on tumor growth rather than the determinants of metastatic spread [1].

One of the rate-limiting steps in breast cancer metastasis is dissemination of primary tumor cells into the bloodstream to yield circulating tumor cells (CTCs) [3]. For CTCs to invade and recolonize in distant tissues, cells must successfully survive the inhospitable and challenging environment of the circulatory system [1, 3]. Due to the scarcity of CTCs in blood samples and heterogeneity in both phenotype and metastatic potential, CTCs are inherently difficult to detect and target [3]. Yet, breast cancer patients with detectable CTCs have a significantly higher risk of metastatic recurrence and death [4]. Pioneering clinical studies showed that neoadjuvant treatment of breast cancer patients before surgery with drugs aimed at reducing tumor growth actually led to a greater than 1000-fold increase in CTCs and reduced relapse-free survival [5]. More recent pre-clinical studies now show that neoadjuvant chemotherapy induces blood vessel bursting, CTC shedding and increases the risk of metastasis [6]. Importantly, this therapy-induced CTC metastasis resulted from chemotherapy drugs with highly divergent mechanisms for suppressing cell growth, including microtubule stabilization and DNA damage, demonstrating that a broad range of existing chemotherapies could inadvertently have metastasis-promoting effects [6].

Given the widespread use of neoadjuvant chemotherapy in breast cancer, it is therefore important to identify therapies that could reduce the metastatic potential of CTCs which may be currently overlooked by drug development pipelines and clinical trials focused primarily on tumor growth. Triple-negative breast cancer (TNBC) poses a particular clinical challenge, as this disease rapidly progresses to metastasis. TNBC does not express the molecular markers that are impacted by targeted therapies against estrogen signaling or HER2 [7], so chemotherapy and radiation are the primary remaining options. Furthermore, TNBC patients are just generally recommended for chemotherapy by the current National Comprehensive Cancer Network (NCCN) guidelines, without any specific guidance on which therapies could reduce metastatic risk [8]. While the 2020 FDA-approval of an antibody–drug conjugate (sacituzumab govitecan-hziy) for metastatic TNBC indicates progress with targeted therapies [9], there remains a need to improve treatment options for patients with early TNBC to reduce metastatic risk.

During dissemination to distant tissues, tumor cells inevitably encounter non-adherent environments, such as the bloodstream or lymphatics [10]. Our group has demonstrated that breast tumor cells in non-adherent microenvironments form unique microtentacles (McTNs) on their surface that promote reattachment to endothelial cells [11]. These McTNs are supported by a coordination of vimentin intermediate filaments and microtubules stabilized with post-translational modifications, such as detyrosination or acetylation of α-tubulin [12, 13]. Moreover, disrupting McTNs through four separate genetic mechanisms reduced experimental metastasis of CTCs in mouse models [14–18]. Since microtubules are a common drug target for cancer therapies [19], we sought to determine if McTNs could be used as a rapid indicator of anti-microtubule drug response and test the principle that targeting microtubules could selectively reduce the metastatic potential of circulating TNBC cells.

## Methods

### Cell culture and chemical compounds

MDA-MB-231, MDA-MB-436 and BT-549 cells were obtained from American Type Culture Collection (ATCC; Manassas, VA, USA) and cultured in DMEM (Corning, Manassas, VA, USA) supplemented with 10% FBS (Atlanta Biologicals, Flowery Branch, GA) and 1% penicillin/streptomycin (PS) (100 μg/ml) (Gemini, Sacramento, CA). MDA-MB-231 GFP cells were infected with a luciferase retrovirus created using pMSCV-Luciferase PGK-hygro expression vector (Addgene, plasmid# 18782 Cambridge, MA) [14, 15] and supplemented with Hygromycin (2.5 μg/ml) to select a stable luciferase expressing cell line. MDA-MB-436 and BT-549 cells were infected with a luciferase GFP lentivirus created using pLL-CMV-rFLuc-T2A-GFP-mPGK-Puro vector (SystemBio, Palo Alto, CA). The MDA-MB-436 and BT-549 cell lines expressing GFP and luciferase were maintained in DMEM/10%FBS/1%PS with 0.5 μg/ml and 0.4 μg/ml of puromycin, respectively.

To create the Tumor Derived (TD) cell lines, Athymic Nude mice were inoculated with the MDA-MB-231 GFP/luciferase, MDA-MB-436 GFP/luciferase, and BT-549 GFP/luciferase expressing cells as described above [14] and allowed to grow for approximately 35 days for the MDA-MB-231 cells, 72 days for the MDA-MB-436 cells, and 97 days for the BT-549 cells or until a 700 mm^3^ tumor formed. The tumors were resected and homogenized, and the cells were then maintained in DMEM/10% FBS/1% PS supplemented with Hygromycin (2.5 μg/ml) to create the MDA-MB-231 Tumor Derived cells (MDA-MB-231 TD), 0.5 μg/ml puromycin to create the MDA-MB-436 Tumor Derived cells (MDA-MB-436 TD), and 0.4 μg/ml puromycin to create the BT-549 Tumor Derived cells (BT-549 TD). All cells were maintained at 37 °C with 5% CO_2_.

### Drug

Vinorelbine Tartrate (solution for in vitro and in vivo experiments respectively, concentration 10 µM, and 5 mg/ml) was provided by BioVision (#1957). For animal experiments, it was freshly diluted in 0.9% sodium chloride on each injection day and injected intravenously (5 mg/kg of body weight).

### Population live cell imaging and McTN scoring

Cells were trypsinized and suspended in DMEM without phenol red and added to low attach plates (Corning) in the presence of Vinorelbine (10 μM) or DMSO (0.1%) and maintained at 37 °C with 5% CO_2_ for 1 h. Live single cells were scored blindly for McTNs, where cells with two or more McTNs extending greater than the radius of the cell body were scored as positive. Populations of 100 or more cells were counted for each replicate, with a minimum of 300 cells counted per condition per experiment.

### Microfluidic cell tethering technology

For live, tethered cell experiments, TetherChip devices with the addition of lipid moieties (DOTAP) were utilized to tether tumor cells to these surfaces (Ibidi µ-Slide I Luer 0.8, Ibidi, Germany) while preventing tumor cell adhesion. [20, 21]. CellMask-Orange stained cells ± Vinorelbine were allowed to tether for 30 min at 37 °C with 5% CO_2_. For fixed, tethered cell experiments there was only 1, baked PEM layer and cells were instead stained with WGA after 3.7% formaldehyde fixation.

### Confocal microscopy

All imaging was conducted on an Olympus FV-1000 confocal at 60 × magnification. For videos of live, suspended cells, a set of five 0.5 μm/slice z-stacks were calibrated to where the cell diameter was largest along the z-axis and imaged every 6.5 s for a total time series of 20 z-stacks. For fixed, tethered cells, z-stack slices were 1 μm thick along the entire thickness of the cell for each timepoint.

### Image analysis

All image processing was conducted on max intensity across the z-axis. For live cell image analysis, a population of 25 cells per condition was analyzed for each individual experimental replicate. For cell isolation and fixation analysis a total of 78 cells in the vehicle control population and 80 cells for the Vinorelbine treated population were analyzed. The data set includes 3 experimental replicates total. For further metrics, outlines of the cell body and full cell outline including McTNs were extracted. For the cell body outline, a combination of built-in MATLAB functions for Otsu thresholding and morphological operators were used [22]. Any cell with a cell body’s centroid that migrated greater than 5 μm was excluded from this study.

The analysis used for the full cell outline with McTNs incorporated a recently designed MATLAB protocol specifically for this purpose [22]. McTN outline analysis is a conglomerate of algorithms optimized for 3 distinct regions: a cell center region to avoid an annular outline that uses a brute force technique of blurring and eroding; for the base of the McTNs, a combination of retinal segmentation and global curvature techniques; for the actual McTNs, a combination of retinal segmentation algorithms and several iterations of rotating anisotropic filtering was executed to optimize for the long filamentous structure [22]. From the cell body and full cell outlines, a max local curvature computation extracted the tips of the McTNs to compute number and distance from McTN tip to cell body, which computed length.

### McTN analysis statistics

All statistics results were measured in MATLAB. Normalness of data distribution was tested for skewness of ± 2 or a kurtosis between 0–6. For normally distributed data, a standard t-test was conducted for 2-sample comparison (tethered verses suspended) and an ANOVA analysis for multi-sample comparisons (drug studies). For non-normally distributed data, an additional Kolmogorov–Smirnov test was conducted for verification.

### Viability assay

Cells were seeded in triplicate at 5 × 10^3^ cells per well for each concentration in a tissue culture treated 96-well plate in DMEM media with phenol red, 100 units/ml penicillin–streptomycin and antibiotic selection media (Hygromycin 2.5 μg/ml, and Puromycin 0.4 μg/ml or 0.5 μg/ml). At time 0, 24 and 48 h after drug treatment, CellTiter96 Aqueous One Cell Proliferation Solution (Promega, Madison, WI) was added, cells were incubated at 37 °C for 2 h, and the absorbance was determined at 490 nm. For 24 h and 48 h, the absorbance was normalized to the absorbance at time 0 to determine cell viability for each drug concentration. Viability was measured as a percent above cell number at time 0 ([(absorbance at day x/absorbance at time 0) − 1] × 100). All values are shown as mean ± SD of triplicate samples.

### Immunofluorescence

Cells were grown on glass coverslips and then fixed in 100% cold methanol (10 min, 4 °C). Immunofluorescence staining was performed as previously described [10] with the following antibodies: Anti-acetyl-α-tubulin (Lys40) (D20G3) XP Rabbit mAb (1:1000; Cell Signaling), Anti-detyrosinated-α-tubulin (1:1000; Abcam), Monoclonal Anti-detyrosinated- α-tubulin (1:10,000: RevMAb), Monoclonal Anti-α-tubulin Clone DM1A (1:5000; Sigma Aldrich) and β-actin (1:2000; AC-15, Sigma). Anti-IgG antibodies conjugated to Alexa-488 (1:1000; Molecular Probes) were used for secondary detection and Hoescht 33342 (1:5000; Sigma) was used for nuclear staining. Images were acquired using an Olympus FV1000 laser scanning confocal microscope (Olympus, Center Valley, PA). The deTyr-Tubulin antibody (RevMab) was developed by Takashi Hotta and Ryoma Ohi [23] and is now commercially available through RevMab under catalogue number RM444 (https://www.revmab.com/index.php/product/anti-detyrosinated-alpha-tubulin-rabbit-monoclonal-antibody-clone-rm444/).

### Immunoblotting

Subconfluent MDA-MB-231 TD, MDA-MB-436 TD, BT-549 TD cells were treated with either Vinorelbine, vehicle control, or Staurosporine. All cells, any suspended and attached, were harvested, lysed with a RIPA lysis buffer, and analyzed by immunoblot as previously described [24] with the following antibodies: Anti-Poly (ADP-ribose) polymerase (PARP; 1:1000, SCBT), Anti-acetyl-α-tubulin (Lys40) (D20G3) XP Rabbit mAb (1:1000; Cell Signaling), Anti-detyrosinated- α-tubulin Monoclonal (1:10,000: RevMAb) or Anti-detyrosinated-α-tubulin (1:1000; Abcam), Monoclonal Anti-α-tubulin Clone DM1A (1:2000; Sigma Aldrich) and β-actin (1:5000; AC-15, Sigma).

### Cell-electrode impedance attachment assay

Real-time, dynamic monitoring of cellular reattachment from suspension was measured using the xCelligence RTCA-DP real-time sensing device (Roche Applied Science) to compare attachment rates of cells pretreated with Vinorelbine or vehicle.

MDA-MB-231 TD, MDA-MB-436 TD, and BT-549 TD cells (20,000 cells/well) were seeded in quadruplicate into 96-well microelectronic sensor standard plates (E-plates) that contained each condition respectively. Attachment was expressed as a change in cell index, an arbitrary unit reflecting the relative change in electrical impedance from cell-electrode interaction across microelectronic sensor arrays. Impedance was measured every 5 min, for a 24-h time period. Values expressed are the mean ± SD of quadruplicate wells.

### Vinorelbine cluster assay

MDA-MB-231 TD, MDA-MB-436 TD, and BT-549 TD cells were treated with Vinorelbine (10 μM) or DMSO (0.1%) and maintained at 37 °C with 5% CO_2_ for 1 h. Cells were counted and diluted (20,000 cells/well) in the presence of Vinorelbine or DMSO in a low-attach 96 well plate to allow for aggregation. At each respective time point, cells were collected and tethered onto a PEM + Lipid coated microfluidic device and incubated at 37 °C for 30 min. Cells were subsequently fixed using 4% formaldehyde and stained with Hoechst 33258 (1:5000). Images were acquired using the Nikon Eclipse Ti-E inverted microscope at 4× magnification.

### Computational cluster analysis

Cluster analysis was performed on previously captured images described above using custom MATLAB scripts (available at https://github.com/ScientistRachel/CellAggregationAnalysis). After smoothing the images with a Gaussian filter (standard deviation of 2 pixels), a series of Laplacian of Gaussian filters ranging in size from 4 to 25 pixels in diameter were applied to each image to detect edges. A maximum projection across filter sizes was binarized using a threshold of filtered intensity greater than 50. The binarized image was morphological closed with a disk of radius 2 pixels, and holes in the objects were filled. Objects smaller than 76 pixels^2^ (a size smaller than a single nucleus) were considered cellular debris and removed from further analysis. Objects larger than 76 pixels^2^ were defined as clusters.

From the binarized images, we report a measured cluster efficiency metric by comparing the number of individual clusters over time. Values at *t* = 0 h were divided by respective experimental final cluster numbers (*t* = 4 h) for each condition. Error bars indicate the standard deviation across experiments. A paired *t*-test was calculated on the logarithm of the cluster efficiencies for the difference between paired experiments of the DMSO and Vinorelbine treated conditions.

### Treatment and inoculation of breast cancer cells in nude mice

For the MDA-MB-231 TD inoculation, eight-to-twelve-week-old female athymic nude-Foxn1nu mice weighing 19–25 g were obtained from Charles River (Frederick, MD) and fed food and water ad libitum. For the MDA-MB-436 TD and BT-549 TD, eight-to-twelve-week-old female NOD.Cg-Prkdc < scid > /Jmice were used at a comparable weight. The mice were maintained in accordance with Institutional Animal Care and Use Committee procedures and guidelines under an approved protocol.

For primary tumor formation, animals were treated intravenously with 5 mg/kg of Vinorelbine Tartrate 24 h and again at 2 h before cell inoculation. MDA-MB-231 TD cells (1 × 10^6^ cells/ml) were treated with 10 μM of Vinorelbine Tartrate 1 h prior to inoculation in animals. The treated cell suspension was then mixed with injected subcutaneously into the 4th mammary gland on the ventral surface of the abdomen of the female mice. Tumor volumes were measured by external caliper measurements weekly from the initial injection to the experimental endpoint. Tumors were measured along the two longest perpendicular axes in the x/y plane of each xenograft tumor to the nearest 0.1 mm with a digital caliper (Thomas Scientific, Inc.).

Depth is assumed to be equivalent to the shortest of the perpendicular axes (*y*), and volume is calculated according to the: *V* = *xy*^2^/2, as the standard practice for xenograft tumors. Signs of tumor ulceration or maximum tumor volume were recorded during each measurement as an experimental endpoint.

For lung retention and metastatic recurrence in MDA-MB-231 TD cells, animals were obtained and treated with Vinorelbine Tartrate as described above. MDA-MB-231 TD cells (1 × 10^6^ cells/ml) were treated with 10 μM of Vinorelbine Tartrate 1 h prior to inoculation in athymic nude animals and injected intravenously.

To recapitulate comparable malignancies to the MDA-MB-231 TD, MDA-MB-436 TD and BT-549 TD were inoculated using NSG/SCID mice. Animals were treated intravenously with 5 mg/kg of Vinorelbine Tartrate only once 2 h before cell inoculation due to the severe immune deficiency of the host animal. Cells were prepared and injected intravenously as described above, Cells were injected into the tail vein, where the first capillary bed they reach is the lung and is used to test for ability of a CTC to trap in the lung as well as outgrowth and serves as a model for metastasis. Animals were subsequently monitored by bioluminescence over the duration of the study. In compliance with University of Maryland IACUC procedures, mice were humanely euthanized at designated terminal endpoints, including any clinical signs of distress, for each independent experiment.

### Bioluminescence imaging

Luciferase expressing cells were injected subcutaneously into mice as above. At the indicated time points following injection, mice were injected intraperitoneally with Luciferin (150 mg/kg, Perkin Elmer) and returned to their cages for 5 min to allow for biodistribution. Mice were anesthetized with 2% isoflurane gas and imaged at 5-min intervals for the maximum photon emission. Total 60 photon flux (photons/s) was calculated and corrected for tissue depth by spectral imaging using Living Image 3.0 software (Xenogen). Bioluminescence generated by the inoculated breast tumor cell line from each mouse was normalized to the initial maximum flux signal at the time of the experiment from the same mouse. The average light generated by the experimental animals was compared to that of the control animals. Bioluminescence was only detected in viable cells expressing the firefly luciferase gene, indicative of an active metabolism.

### Immunohistochemistry and pathology

At lung saturation exceeding 1000-fold over the initial bioluminescence signal, mice were humanely euthanized. Lung tissue and tumors were removed, fixed in formalin for 24 h, embedded in paraffin wax, and serially sectioned (4-μm thick).

All immunostaining was performed by Mass Histology Services (Worcester, MA). Mass Histology supplied the H&E staining, Ki67 and Human mitochondria antibodies.

### Parsortix spiking and isolation

The Parsortix device was operated using the procedures as previously described [25]. MDA-MB-231TD (1 × 10^3^) cells were spiked into 7.5 mL of whole blood and the vacutainer was attached directly onto the Parsortix device. Cells were harvested after isolation and tethered onto a µ-Slide I Luer 0.8 Ibidi channel slide (Ibidi, Germany) (Cat: 80191) coated with PEM + DOTAP for 30 min. Live cells were stained with CellMask Orange (Thermo Fisher Scientific) (Cat: C10045) and NucBlue Live ReadyProbes Reagent (Thermo Fisher Scientific) (Cat: R37605) to visualize the plasma membrane and nucleus, respectively. Images were acquired using an Olympus IX81 microscope with a Fluoview FV1000 confocal laser scanning system.

#### Vortex spiking and isolation

The Vortex VTX-1 device was operated using the procedures as previously described [43]. MDA-MB-231TD cells (5 × 10^3^) were spiked into 4 mL of whole blood diluted in PBS to a total volume of 40 mL, for a 10X blood dilution. Cells were isolated with the VTX-1 and collected into a 1.5 mL eppendorf tube (~ 300 uL total volume in PBS).

Half of the collected sample (~ 150 uL) was treated with 0.1% DMSO and the other half of the sample was treated with 10 μM Vinorelbine for 1 h in a 96-well low-attach plate (Corning, Corning, NY) (Cat: 3474). Total volume was collected for both samples and tethered onto a µ-Slide I Luer 0.8 Ibidi channel slide (Ibidi, Germany) (Cat: 80191) coated with PEM + DOTAP for 30 min. Cells were then fixed with 3.7% formaldehyde and stained with Hoechst 33258 (1:5000) and Wheat Germ Agglutinin (WGA, Alexa Fluor 594) (1:100) (Thermo Fisher Invitrogen) (Cat: W11262) to visualize the nucleus and cell membrane, respectively. Images were acquired using an Olympus IX81 microscope with a Fluoview FV1000 confocal laser scanning system.

## Results

### Vinorelbine can decrease microtentacles with minimal impact on cell viability

To develop a proof-of-concept study that examined if metastatic dissemination could be selectively targeted compared to primary tumor growth, we prioritized Vinorelbine, a 3rd generation synthetic vinca alkaloid that inhibits microtubule polymerization [26]. Circulating tumor cells produce unique microtentacles (McTNs) on their cell surface that are supported by microtubules and can be inhibited with a range of tubulin-disrupting compounds, including Colchicine and Nocodazole [27]. Vinorelbine has the additional advantages of being FDA-approved for breast cancer treatment, oral bioavailability, and an improved pharmacokinetic profile that extends serum lifetime and reduces toxicity for hematologic and neuronal cells [26].

Molecular profiling has also subdivided triple-negative breast cancer into 4 subtypes (TNBC type-4) which have distinct clinical outcomes, with the mesenchymal (M) subtype showing by far the highest frequency of lung metastasis in breast cancer patients (46%) [7]. We examined multiple basal-like triple-negative breast cancer lines including the MDA-MB-231, MDA-MB-436 and the BT-549. However, the MDA-MB-231 TNBC cell line presents the M-subtype profile and is more often used for studies of experimental lung metastasis in mice [28]. To test the effects of Vinorelbine treatment on CTC lung metastasis, we prioritized tumor-derived MDA-MB-231 cells (MDA-MB-231 TD), which express luciferase for whole-animal optical imaging, for our long-term study over the MDA-MB-436 and BT-549 cell lines. The MDA-MB-231 TD cell line produced higher observed McTNs scores that correlated with enhanced tumor cell reattachment.

**Fig. 1:**
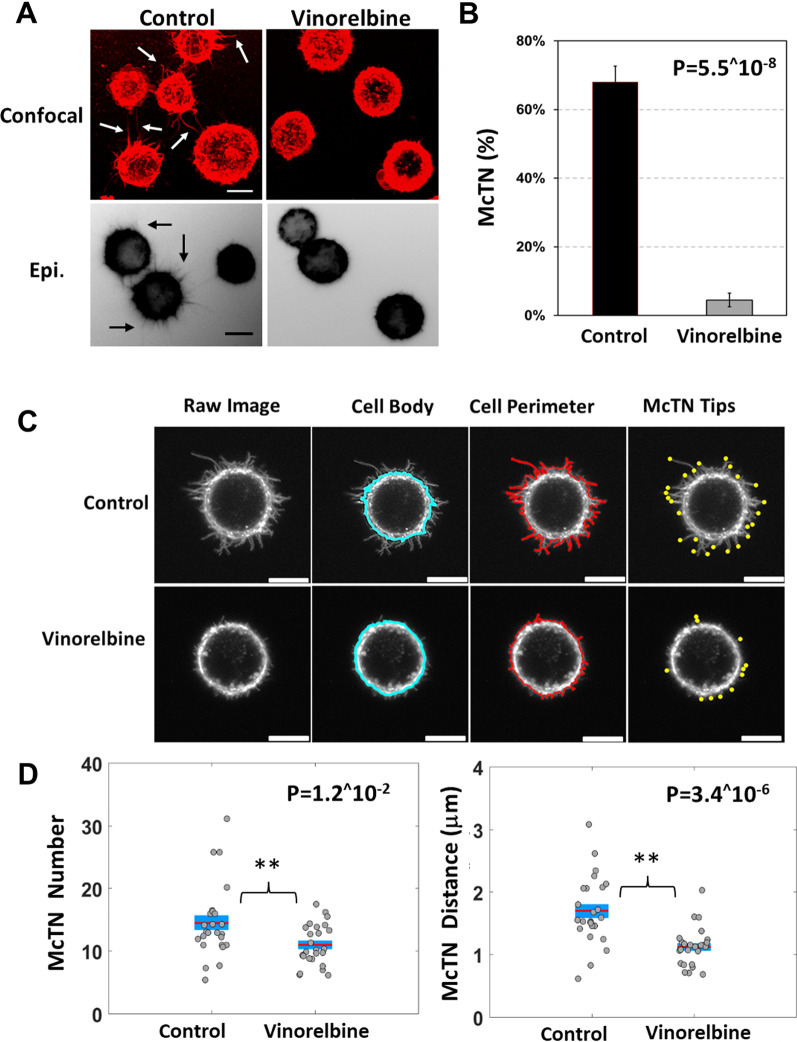
Vinorelbine decreases McTNs on breast tumor cells. MDA-MB-231 TD cells stained with CellMask Orange cell membrane dye, detached and suspended in media containing vehicle (0.1% DMSO) or Vinorelbine (10 μM) for 1 h. **a** Representative confocal images (top) and inverted epifluorescence images (bottom) of free-floating cells on a low attach plate. Scale bars correspond to 10 μm. **b** Vinorelbine caused a significant decrease in McTN frequency (%) compared to vehicle control. McTN scoring consists of mean values from four independent experiments where 100 cells were blindly counted and averaged. **c** Representative raw images of tethered cells and computer determined cell body outline (blue), cell perimeter (red), and McTNs tips (yellow). **d** Live cell analysis measuring an average number of McTN tips for cells treated with vehicle (0.1% DMSO) or Vinorelbine (10 μM) for 1 h. McTN number is the number of McTNs per cell. McTN distance is average distance of McTN tips from cell body boundary. For live cell image analysis, a population of 25 cells per condition was analyzed from 3 independent experimental replicate

Using microfluidic cell tethering technology (TetherChip) that facilitates McTN imaging with confocal microscopy [20, 21] (Fig. 1a, arrows, Additional file 1: Fig. S1A), we determined that McTNs produced by the MDA-MB-231 TD, MDA-MB-436 TD and BT-549 TD cells are strongly inhibited by a one hour treatment of 10 μM Vinorelbine.

The reduction of McTNs was apparent using either confocal microscopy of tethered cells (Fig. 1a, top, Additional file 1: Fig. S1A) or live-cell epifluorescence of free-floating cells (Fig. 1a, bottom). Blinded quantitation (Fig. 1b, Additional file 1: Fig. S1B) demonstrated a statistically significant decrease in the percentage of cells producing McTNs after treatment with Vinorelbine (10 µM, 1 h.) compared to vehicle control (0.1% DMSO, 1 h). However, cell viability remained very high in the MDA-MB-231 TD (78%), MDA-MB-436 TD (74.2%) and BT-549 TD (84.9%) even after a continuous 24-h Vinorelbine treatment (Additional file 2: Fig. S2A, C), despite causing cell rounding and detachment (Additional file 2: Fig. S2B, D).

Given the decreased McTN frequency, we performed a perimeter analysis using Fiji ImageJ (National Institute of Health, MD, USA) to further quantify the McTN frequency of MDA-MB-231 TD, MDA-MB-436 TD and BT-549 TD cells (Additional file 1: Fig. S1C). This allowed us to compare size, circularity and perimeter of the cell membrane using our TetherChip technology to measure differences between the vehicle control treatment and Vinorelbine. As observed with McTN quantification, Vinorelbine significantly reduced the size of the perimeter of the cell after Vinorelbine (10uM, 1 h) compared to vehicle control.

### Computational analysis of Vinorelbine effects on McTN metrics

To more precisely define alterations in McTN characteristics, our research team recently developed a custom image analysis program written in MATLAB [22] that automatically identifies McTN tips (Fig. 1c, Additional file 1: Fig. S1C, yellow circles) by finding the points of maximum local curvature in the cell perimeter trace (Fig. 1c, Additional file 1: Fig. S1D, red lines). This yields an automatic count of McTN number per cell (Fig. 1d, Additional file 1: Fig. S1E, number) and comparison of distance between McTN tips and the cell body (Fig. 1c, Additional file 1: Fig. S1D, blue line), which defines the average McTN tip distance (Fig. 1d, Additional file 1: Fig. S1E, distance). TetherChip technology improves image clarity with confocal microscopy and consequently a more accurate computational analysis by reducing the drift of non-adherent tumor cells [22]. This enables high-resolution quantitative imaging of tumor cells in a non-adherent microenvironment that better simulates the conditions of metastatic dissemination compared to cells adhered on rigid surfaces or extracellular matrix. One hour of Vinorelbine treatment significantly reduced the average number and length of McTNs on MDA-MB-231 TD cells (Fig. 1d), as well as MDA-MB-436 TD and BT-549 TD cells (Additional file 1: Fig. S1E). A statistical 90% power analysis reveals statistically significant drug responses of McTN tip distance using as few as 21 cells and also identified that Vinorelbine significantly reduced McTN length, as compared to McTN number per cell which would require 61 cells.

The ability to yield quantitative McTN metrics from such small numbers of tumor cells increases the feasibility of applying these methods to CTCs, where cell numbers are often very limited [3]. Moreover, since this drug response can be measured in only one hour, without requiring growth, the longer-term pressures of selection and adaptation that occur during in vitro tumor cell propagation can be reduced.

### Vinorelbine increases tubulin acetylation but rapidly disrupts the microtubule network

**Fig. 2:**
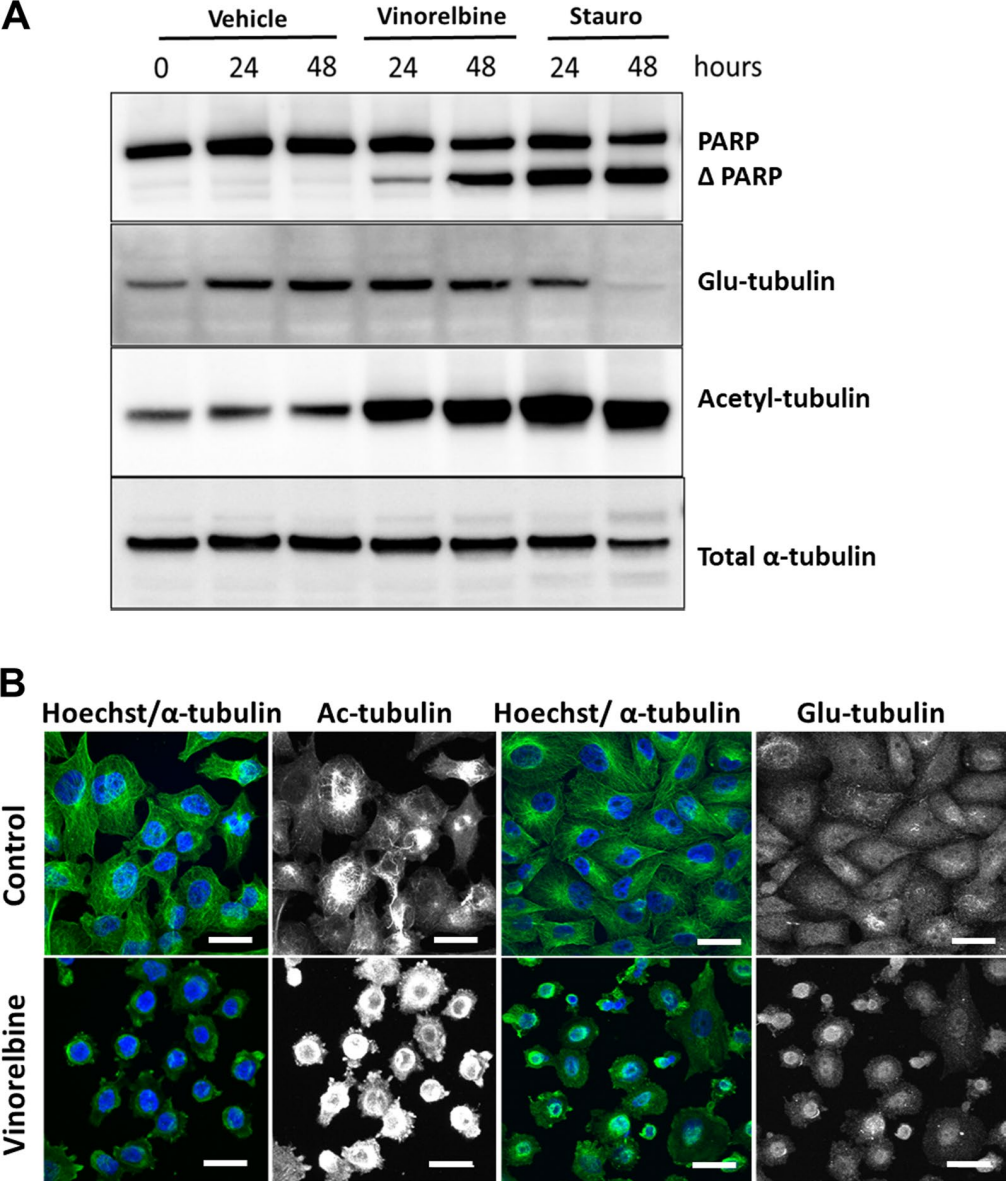
Vinorelbine increases acetylated α-tubulin while decreasing filamentous microtubule network. **a** Immunoblot analysis of Vinorelbine (10 μM) treated MDA-MB-231 TD cells results in an increase in acetylated α-tubulin (acetyl-tubulin) whereas detyrosinated tubulin (Glu-tubulin) and total α-tubulin remain unchanged at 24 h and 48 h, compared with vehicle control (0.1% DMSO). Vinorelbine (10 μM) yielded minimal PARP cleavage at 24 h. However, by 48 h., approximately 50% PARP cleavage is observed. **b** Immunofluorescence shows filamentous tubulin (green) is destroyed within 15 min of 10 μM Vinorelbine treatment, as well as the organization of acetylated (Ac) and detyrosinated (Glu) microtubules. Analysis at additional times (data not shown) reveals the continued absence of filamentous tubulin. Hoechst was used to visualize the nuclei (blue). Images taken at 60 × magnification. Scale bar = 20 µm

McTNs are enriched in stabilized microtubules that have been post-translationally modified with either acetylation or detyrosination of α-tubulin [12, 13, 27]. Immunoblotting demonstrated that Vinorelbine treatment did not detectably alter α-tubulin compared to vehicle-treated control cells, in the MDA-MB-231 TD, MDA-MB-436 TD and BT-549 TD cells (Fig. 2a, Additional file 3: Fig. S3A). However, α-tubulin acetylation (Acetyl-tubulin) was unexpectedly strongly increased by Vinorelbine treatment (Fig. 2a), while remaining consistent in MDA-MB-436 TD and BT-549 TD cells (Additional file 3: Fig. S3A). Surprisingly, Vinorelbine treatment did not show an increase in detyrosination (Glu-tubulin) in MDA-MB-231 TD, MDA-MB-436 TD and BT-549 TD cells at 24 h and 48 h post treatment cells (Fig. 2a, Additional file 3: Fig. S3A).

Despite this increase in acetylation of total α-tubulin protein in the MDA-MB-231 TD cells, the microtubule network was rapidly disrupted by Vinorelbine as early as 15 min after treatment, and the increased levels of acetylated α-tubulin did not form discrete filaments (Fig. 2b). Furthermore, this loss in the filamentous microtubule network was also observed in MDA-MB-436 TD and BT-549 TD cells treated with Vinorelbine (Additional file 3: Fig. S3B) with a collapse of both acetylated α-tubulin and detyrosinated α-tubulin.

While the overall viability of the MDA-MB-231 TD, MDA-MB-436 TD and BT-549 TD cells is only minimally impacted by continuous Vinorelbine treatment for either 24 h or 48 h (Additional file 2: Fig. S2A, C), we immunoblotted for an earlier apoptotic marker (PARP cleavage) to determine how short-term Vinorelbine treatment affected apoptotic signaling. PARP cleavage following Vinorelbine remained minimal at 24 h, but cells continuously treated for 48 h displayed higher levels of PARP cleavage, compared to vehicle control cells, indicating apoptotic commitment (Fig. 2a, Additional file 3: Fig. S3A). To focus on mechanisms aside from cell death, we targeted the Vinorelbine treatment window for subsequent functional tests between the 1 h timepoint shown to reduce McTNs (Fig. 1, Additional file 1: Fig. S1) and the 24 h timepoint where cell viability remains nearly 90% (Additional file 2: Fig. S2A, C) and apoptosis minimal (Fig. 2a, Additional file 3: Fig. S3A).

### Tumor cell reattachment and homotypic aggregation are decreased by Vinorelbine treatment

**Fig. 3:**
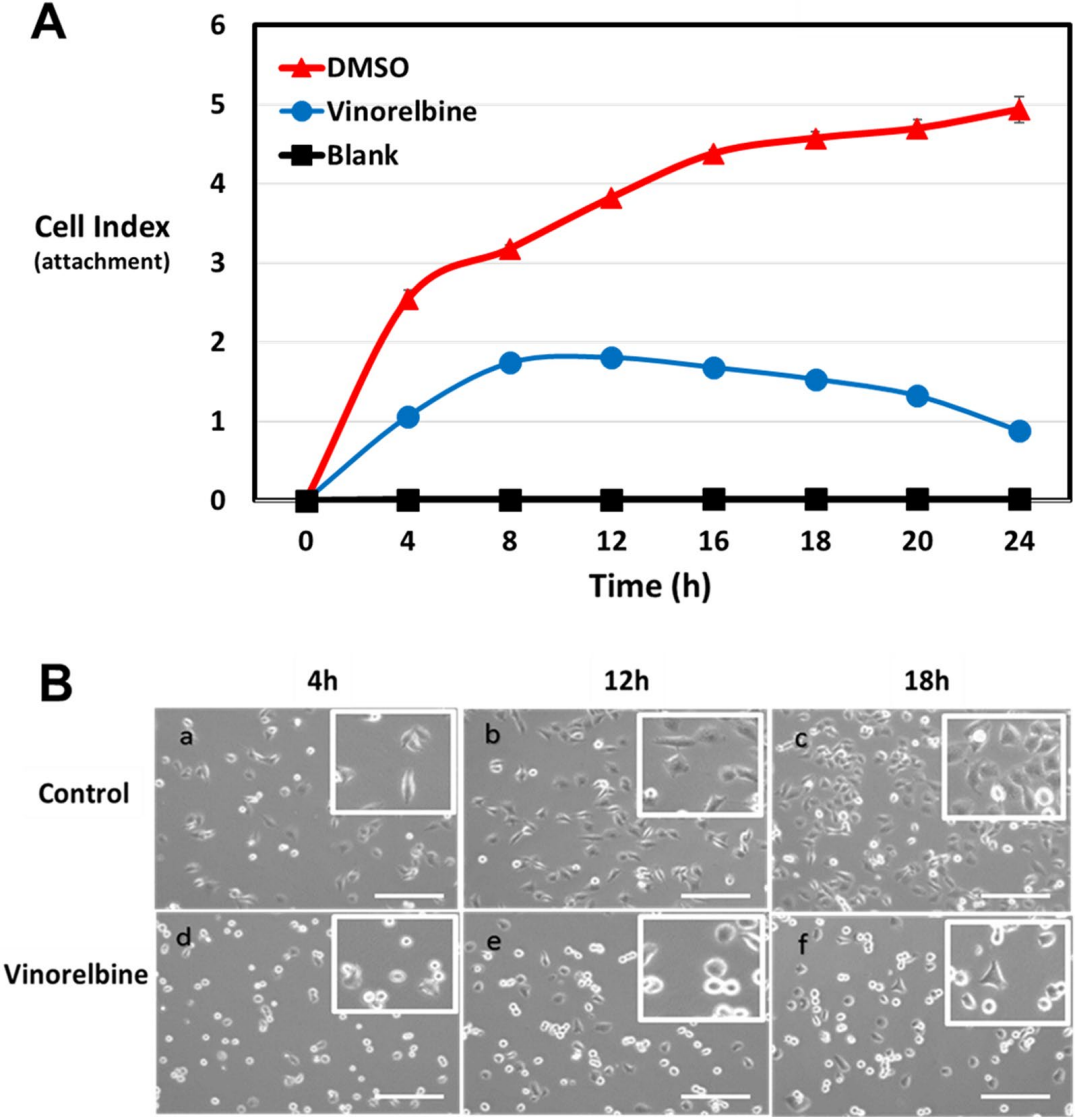
Vinorelbine treatment decreases tumor cell reattachment. **A** Reattachment efficiency of the MDA-MB-231-TD cells treated with Vinorelbine (10 μM) is significantly lower than vehicle control treated cells (0.1% DMSO). Changes in impedance are apparent as early as 1 h and significant differences continue for 24 h after initial seeding. Representative experiment from three independent experiments; each performed in quadruplicate. **B** Representative phase contrast images of vehicle control (a–c) and Vinorelbine-treated (d–f) MDA-MB-231-TD cells over time to visualize cell attachment or lack of attachment (rounding). Panels, 4 × magnification; Insets, 10 × magnification. Scale bar = 100 µm

We have previously shown that McTNs are tubulin-based structures that promote endothelial cell reattachment [11]. Independent macroscopic studies have also determined that tubulin is more important than actin for metastatic reattachment of CTCs in vivo [29]. Pre-treatment of MDA-MB-231 TD, MDA-MB-436 TD, BT-549 TD cells with Vinorelbine for 1 h reduced tumor cell reattachment in vitro, as measured by real-time electrical impedance (Fig. 3A, Additional file 4: Fig. S4A). Phase contrast images reinforce that Vinorelbine-treated cells do not attach as efficiently as vehicle-treated cells (Fig. 3B, Additional file 4: Fig. S4B). The vehicle-treated population began to reattach and spread by 1 h and the majority were completely attached at 18 h., while cells treated with Vinorelbine remained rounded and only minimally attached after 18 h. Thus, the effects of short term Vinorelbine can disrupt the microtubule network (Fig. 2b, Additional file 3: Fig. S3B) and inhibit McTNs (Fig. 1, Additional file 1: Fig. S1), leading to reduced tumor cell reattachment within a 24 h window when cell viability and apoptosis are not significantly affected (Fig. 2a, Additional file 2: Fig. 2A, C, Additional file 3: Fig. S3A).

**Fig. 4:**
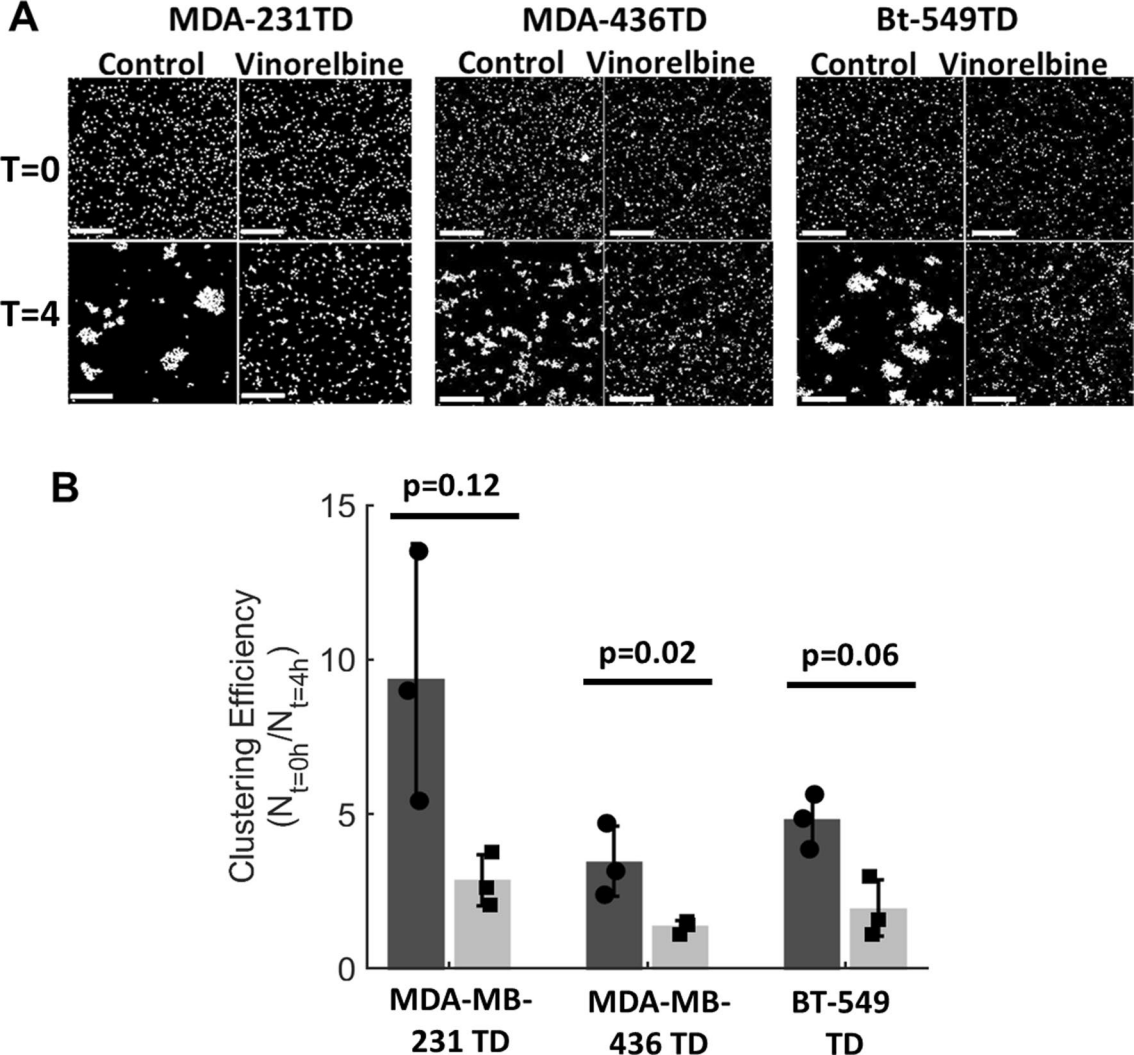
Vinorelbine treatment decreases homotypic cluster aggregation in vitro*.* MDA-MB-231 TD, MDA-MB-436 TD and BT-549 TD cells were treated in attached conditions for 1 h in Vinorelbine (10 μM). Cells were trypsinized, counted, and plated in a low attach plates for 4 h to test aggregation. At given timepoints, cells were tethered onto a TetherChip surface and stained with the nuclear dye Hoechst 33258 (1:5000). **a** Representative images of control and Vinorelbine-treated MDA-MB-231 TD, MDA-MB-436 TD and BT-549 TD cells over time to visualize cluster formation efficiency. Images taken of tethered and stained cells at *t* = 0 and 4 h. at a 4 × magnification. Scale bar = 100 µm. **b** Analysis measuring the efficiency of clustering by comparing the number of individual clusters over time. Individual values at *t* = 0 h were divided by respective experimental final cluster numbers (*t* = 4 h) for each condition

Beyond endothelial attachment, McTNs promote homotypic tumor cell aggregation [17, 30], and recent studies show that CTC clusters have up to 50-fold higher metastatic efficiency [31, 32]. Since McTNs are strongly inhibited by Vinorelbine (Fig. 1, Additional file 1: Fig. S1), we sought to investigate whether treatment with Vinorelbine could disrupt the formation of tumor cell clusters in a non-adherent microenvironment. As observed with reattachment, Vinorelbine was effective in reducing the ability of non-adherent MDA-MB-231 TD cells to form clusters over time (Fig. 4). Visualized with Hoechst staining, both the vehicle-treated and Vinorelbine-treated MDA-MB-231 TD, MDA-MB-436 TD and BT-549 TD cells exhibited similar initial single-cell densities in suspension (Fig. 4a, *top panels*). While the vehicle control in each cell line formed large homotypic aggregates during 4 h in non-adherent conditions, Vinorelbine treatment (10 μM) strongly reduced the formation of tumor cell clusters, (Fig. 4a, *bottom panels (t* = *4 h)*, Additional file 5: Fig. S5).

To objectively analyze the formation of individual clusters, a custom MATLAB script was written to count aggregates after Vinorelbine treatment (Fig. 4b). In each experimental replicate, cluster counts were compared to each respective experimental starting cluster number (*t* = 0). Any object larger than 76 pixels^2^ (the size of a single nucleus) was counted and analyzed by taking a clustering efficiency ratio (*t* = 0/*t* = 4). A paired t-test between the vehicle control population and Vinorelbine-treated conditions in the MDA-MB-231 TD (*p* = 0.12), MDA-MB-436 TD (*p* = 0.02) and BT-549 TD (*p* = 0.06) all reduced clustering efficiency (Fig. 4b), that paralleled the microscopic observations (Fig. 4a).

### Vinorelbine treatment for 24 h does not significantly improve primary tumor survival

**Fig. 5:**
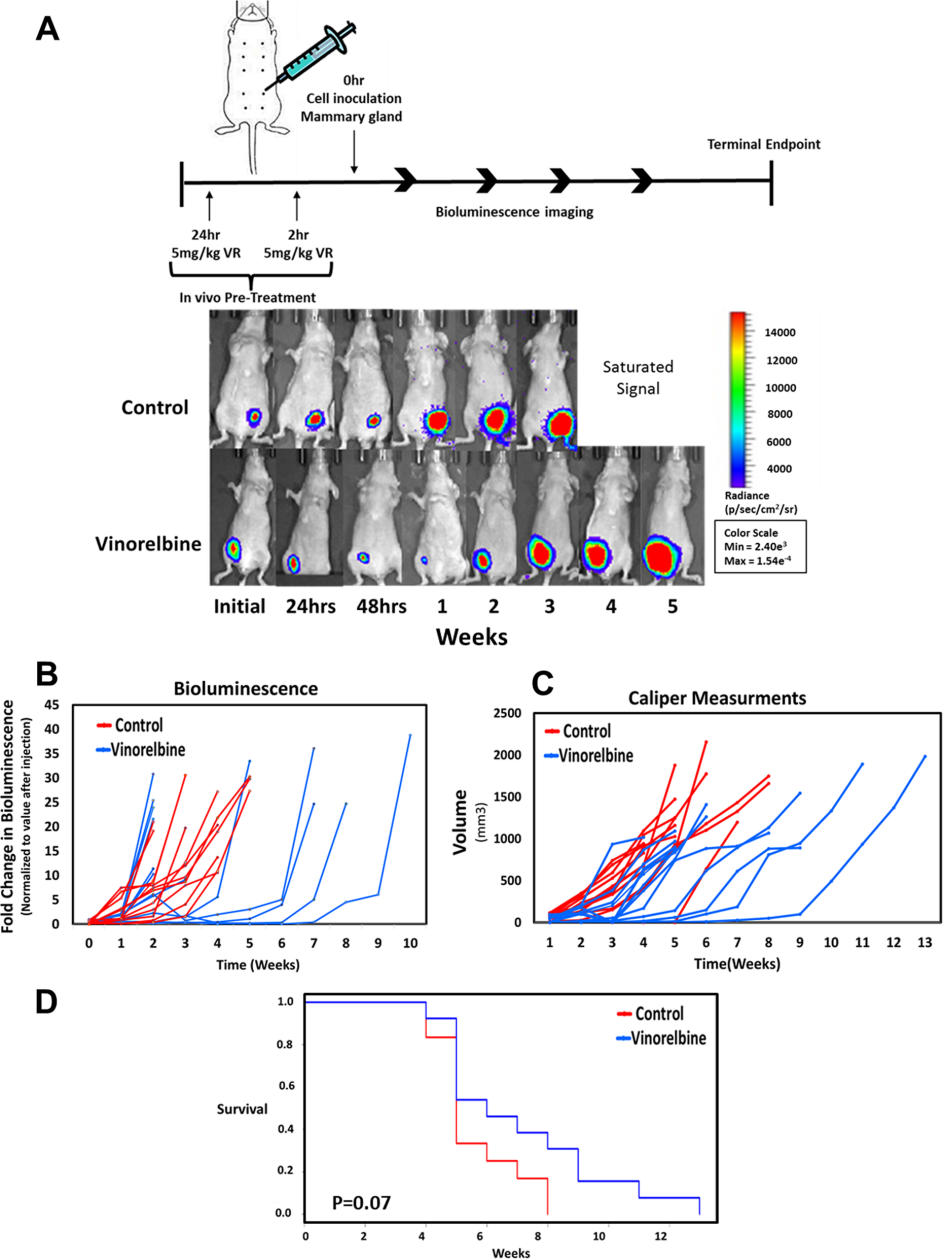
Primary tumor development is uninhibited in the presence of Vinorelbine. **a** Representative bioluminescence imaging from mice treated with vehicle control (0.1% DMSO) or 5 mg/kg Vinorelbine during a 24 h and 2 h prior to injection of MDA-MB-231 TD cells. Photon flux color scale is shown to the right. **b** Graphical representation of the growth curve in each mouse measured as a fold difference of bioluminescence signal over time. To quantitate values for each time point, the background was subtracted from the peak signal, and the difference was normalized to the initial value (first timepoint) for each animal (*n* = 5 per group). **c** Graphical representation of the tumor size for each mouse measured by external caliper measurement. Volumetric measurement of xenografted tumors was obtained using the ellipsoid calculation (*V* = *xy*^2^/2). **d** The probability of overall survival was assessed by Kaplan–Meier analysis (Log-rank test *P* = .07) for mice treated with Vinorelbine

Like many chemotherapies, the central goal of microtubule disruption with Vinorelbine is to exert an anti-mitotic action on dividing tumor cells and induce cell death [19]. However, the current experiments show that a targeted treatment of Vinorelbine for up to 24 h can reduce McTNs and tumor cell reattachment without significantly affecting overall cell viability or inducing apoptosis. To test whether Vinorelbine treatment in vivo could selectively target metastatic phenotypes (i.e. McTNs) rather than cell growth, we developed a dosing strategy to test differential effects on primary tumor growth and the metastatic efficiency of CTCs (Fig. 5a, top schematic).

Mice received a bolus of intravenous Vinorelbine (5 mg/ml) at two timepoints prior to cell implantation (24 h and 2 h prior) while MDA-MB-231 TD cells were treated with Vinorelbine for 1 h prior to injection. This dose remains well-below the maximum tolerated dose for Vinorelbine in mice (12 mg/kg) [33], especially since Vinorelbine is more than 90% cleared from the bloodstream within 24 h [26]. Calculation of human-equivalent dose yields 10.9 mg/m^2^ in the treated mice, which also remains significantly below the standard I.V. dose for Vinorelbine in humans (25–30 mg/m^2^) [34].

To test the effects on primary tumor growth with this dosing strategy, a suspension of drug-treated cells was injected into the mammary gland of female NCR-nu/nu mice. Using whole-animal bioluminescence imaging (Fig. 5a, top row (control)), we show early detection of primary tumor growth in all the mice treated with the vehicle control (12/12). In the Vinorelbine treated group, primary tumor growth was slightly delayed (Fig. 5a, bottom row (Vinorelbine)), but tumors rapidly regrew in all mice reaching similar volume and radiance to that of control (13/13). Quantitative measurements via either bioluminescence (Fig. 5b) or caliper measurements (Fig. 5c) show that the short treatment with Vinorelbine yielded on average a 3–4 week delay in primary tumor growth. However, Kaplan–Meier survival analysis (Fig. 5d) showed that the clinical endpoints did not reach statistical significance (Log-Rank *p *= 0.0697). Therefore, a targeted window of Vinorelbine treatment could slightly delay primary tumor growth, but overall survival was not significantly improved.

### Vinorelbine reduces metastasis more effectively than primary tumor growth

**Fig. 6:**
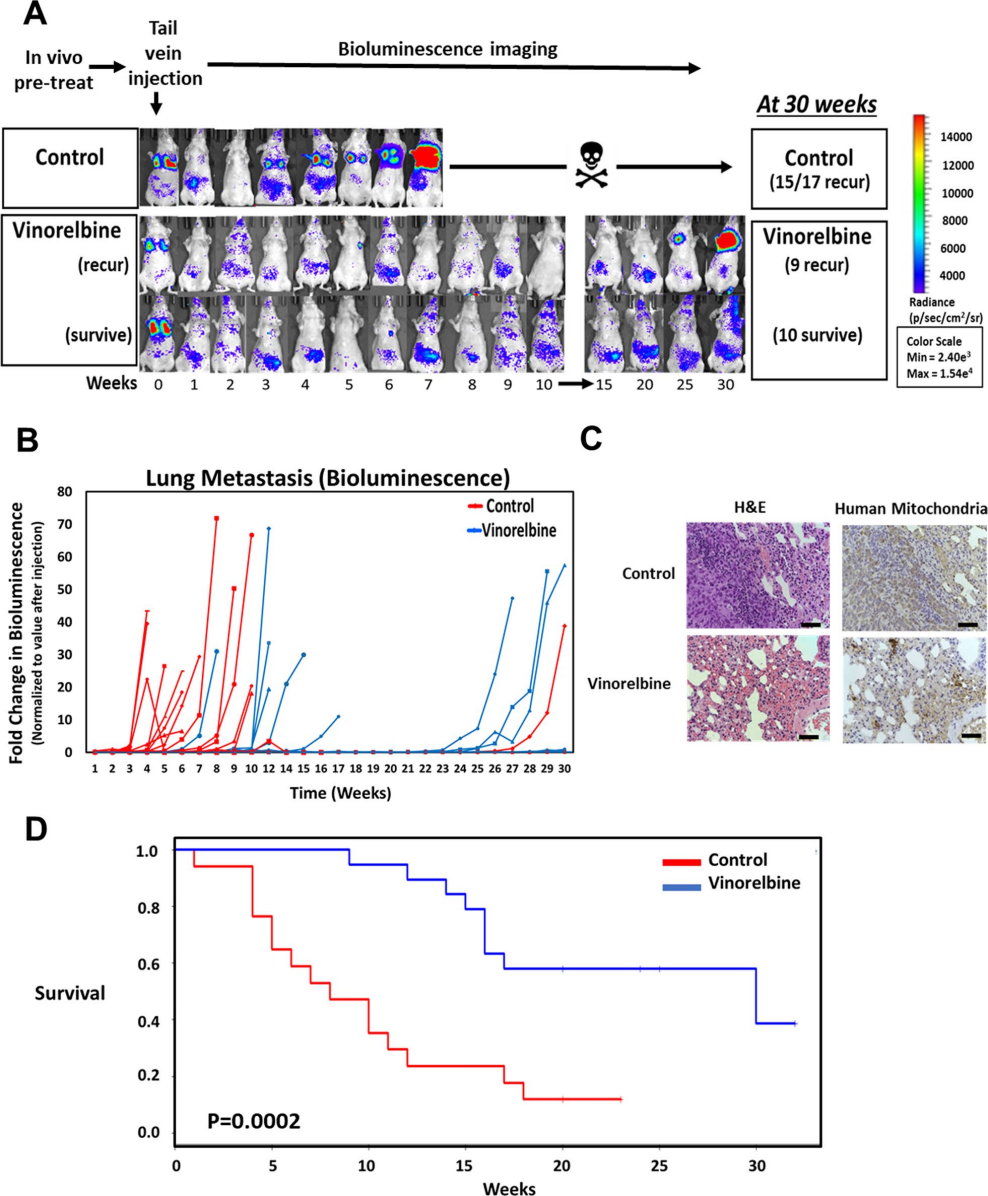
Lung retention and metastatic development after Vinorelbine treatment. **a** Representative bioluminescence images of mice treated with vehicle control (0.1% DMSO) or 5 mg/kg Vinorelbine at 24 h and 2 h prior to injection of MDA-MB-231 TD cells. The vehicle control population resulted in 15 out of 17 animals (88%) exhibiting tumor formation in lung tissue between 3–12 weeks post inoculation with 2/17 (12%) surviving. The focused Vinorelbine treatment resulted in 10 out of 19 animals (53%) with disease-free survival at 30 weeks and 9 out of 19 animals (47%) with delayed tumor formation. Photon flux color scale is shown to the right. **b** Fold differences of retained bioluminescence in the lung of each mouse inoculated with MDA-MB-231 TD cells via tail vein. Data represent individual animal examined and measured as a fold change of the initial value of each independent animal. **c** Representative images of immunohistochemistry for human mitochondria and hematoxylin and eosin (H&E) staining performed at ethical end-points. Images captured at a magnification of 20 × indicate metastatic burden, scale bar, 200 μm. **d** Overall survival was assessed by Kaplan–Meier analysis (Wilcoxon test *P* < 0.001 and Log-rank test *P* < 0.001) for mice treated with Vinorelbine

To investigate potential selective effects on CTC metastasis, the mice were treated with the same dosing strategy of Vinorelbine (24 h and 2 h pre-transplantation) prior to injection of MDA-MB-231 TD cells via tail vein. In this experimental metastasis model, Vinorelbine yielded a drastic decline in metastatic recurrence in the lungs (Fig. 6a). Bioluminescence from the injected MDA-MB-231 TD cells was monitored weekly for 30 weeks, and by the end of the time course, only 2/17 animals treated with the vehicle control had survived (12%). In sharp contrast, 10/19 animals treated with the brief 24 h period of Vinorelbine (53%) survived at the end of 30 weeks. Bioluminescence measurements from the individual mice indicated that the bulk of the control animals reached signal saturation within 3–9 weeks (Fig. 6b) while the Vinorelbine treated mice did not show recurrence until much later. Animals that presented clinical signs of distress from metastatic burden (labored breathing, weight loss), or a bioluminescence signal greater than 80-fold over the initial signal were considered to have reached a terminal endpoint and human-specific immunohistochemistry on isolated lung tissue confirmed the presence of metastatic lesions from the injected human tumor cells (Fig. 6c). Strikingly, Kaplan–Meier survival analysis showed the Vinorelbine treated group had a remarkably improved metastatic tumor survival than the control group (Log-Rank *p* = 0.0002, Fig. 6d). As a result of the brief 24 h treatment window with Vinorelbine, median metastatic tumor survival was extended from 8 weeks in the control group to 30 weeks in the Vinorelbine treated group (Fig. 6d).

A similar model was used to determine the efficacy of Vinorelbine in delaying or inhibiting metastatic outgrowth in two additional triple-negative breast cancer cell lines (Additional file 6: Fig. S6). Using NOD/SCID/gamma mice, we examined the effects of Vinorelbine in MDA-MB-436 TD and BT-549 TD cells lines. Due to the severity and immunologic incompetence of the host, mice received only a single bolus of intravenous Vinorelbine (5 mg/ml) twenty-four hours prior to cell implantation, while each cell line was treated with Vinorelbine for 1 h prior to injection. Notably, inhibition of metastatic recurrence in the lung was observed in both the MDA-MB-436 TD and the BT-549 TD cell lines when measured by bioluminescence (Additional file 6: Fig. S6A, C). At 7 weeks, nearly all animals in the MDA-MB-436 TD and BT-549 TD cohorts are currently surviving, however, the bioluminescence between the two conditions is starkly different. The vehicle control treated MDA-MB-436 TD line yielded lung metastasis in 5 out of 5 animals (100%) while the vehicle control BT-549 TD line resulted in lung metastasis in 4/5 (80%) of the mice (Additional file 6: Fig. S6B, D). After 7 weeks, the vehicle control MDA-MB-436 TD mice had a tenfold increase over the initial value, while the BT-549 TD had a twofold increase over the initial bioluminescence value. In contrast, there was not yet recurrence of the MDA-MB-436 TD or the BT-549 TD cells treated with Vinorelbine within the lungs as bioluminescence did not approach or exceed the initial value (Additional file 6: Fig. S6B, D).

### Recovery of live tumor cells from blood samples can rapidly reveal Vinorelbine drug response

**Fig. 7:**
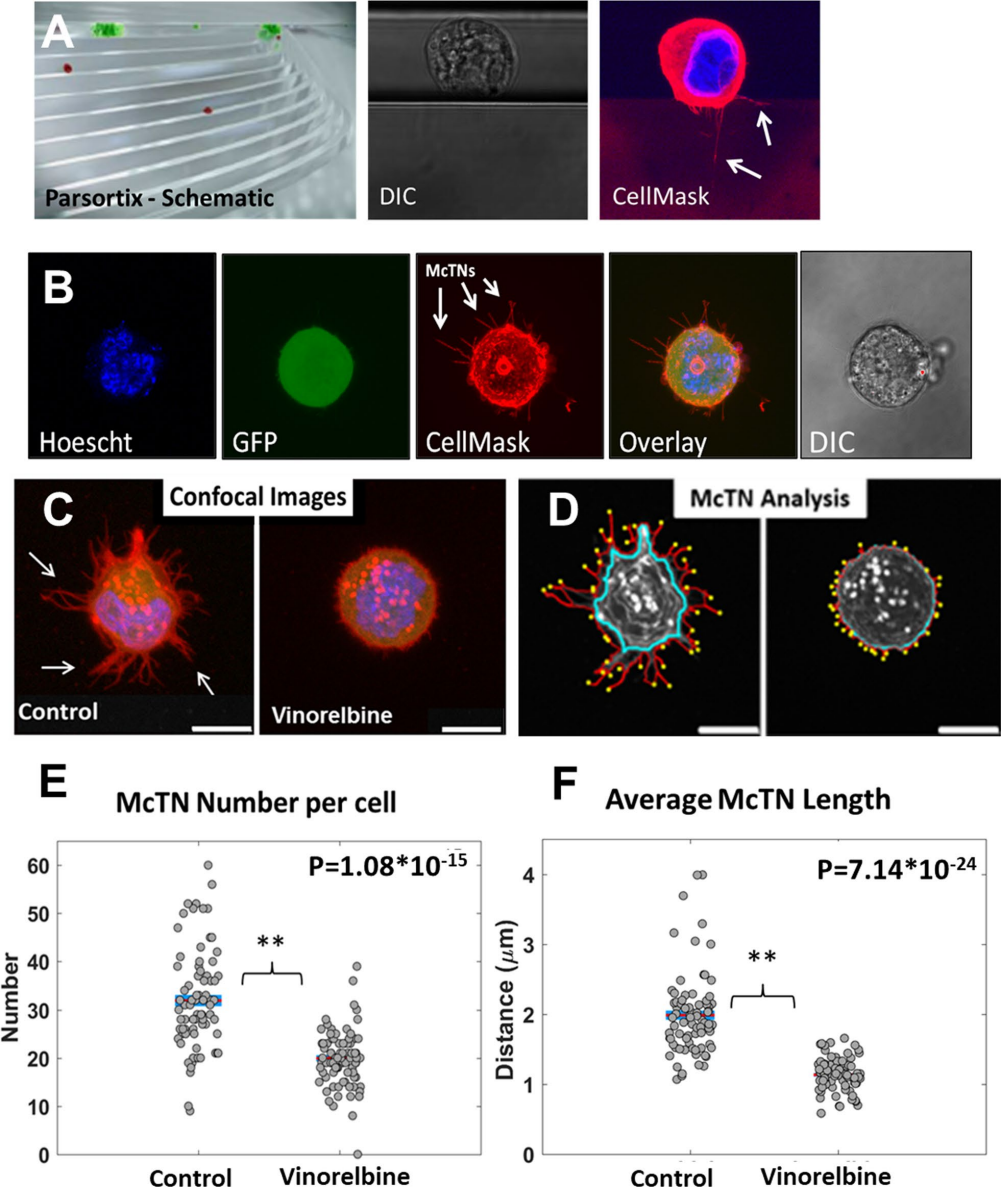
Vinorelbine drug response in tumor cells recovered from blood samples. MDA-MB-231 TD cells captured using ANGLE Parsortix and VTX-1 systems. **a** Parsortix schematic of CTC staircase capture method from whole blood. Live DIC images to visualize cell capture and CellMask orange stained cells to visualize McTNs (arrows). **b** MDA-MB-231 TD cells (1 × 10^3^) spiked into 7.5 ml whole blood were recovered in Parsortix cassette, eluted onto microfluidic cell tethering slides and imaged live after DNA staining with Hoechst and CellMask Orange cell membrane dye. **c** Confocal microscopy of MDA-MB-231 tumor cells (5 × 10^3^) isolated from 7.5 ml whole blood using the VTX-1 system and then treated with Vinorelbine (10 μM, 1 h) or drug vehicle (0.1% DMSO, 1 h) before elution onto microfluidic cell tethering slides, chemical fixation and Hoescht (blue)/WGA (red) staining. Images show an overlay of each color. Arrows show McTN protrusions on isolated tumor cells. **d** McTN analysis of the cell body outline (blue), cell perimeter (red), and McTNs tips (yellow) of fixed and tethered cells. **e** McTN analysis measuring average number McTNs/cell after treatment with vehicle or Vinorelbine. **f** Analysis of average distance of McTN tips from cell body boundary for cells treated with vehicle or Vinorelbine. For cell isolation and fixation analysis a total of 78 cells in the vehicle control population and 80 cells for the Vinorelbine treated population was analyzed from three independent experimental replicates

Patient monitoring in breast cancer patients currently relies on technologies (mammogram, MRI, PET/CT) where a large lesion of more than 10 million tumor cells is required to meet the clinical imaging detection threshold [2]. Consequently, breast cancer patient management and new drug development is focused almost exclusively on tumor growth, rather than metastasis [1]. Given our observations that a targeted Vinorelbine treatment could selectively reduce CTC metastasis, we sought to determine whether this drug response could be monitored rapidly using a more limited number of tumor cells. Several microfluidic technologies have recently been developed allowing for the isolation of live tumor cells from patient blood samples. We used both the ANGLE Parsortix system and the Vortex Biosciences VTX-1 instrument to independently isolate MDA-MB-231 TD cells that had been spiked into standard 7.5 ml or 4 mL human blood samples, respectively. The ANGLE Parsortix uses microfluidic channels where relatively larger tumor cells are trapped at the top of a series of steps, while smaller erythrocytes and leukocytes flow through (Fig. 7a, schematic) [35].

Using live cell confocal microscopy, we show that the MDA-MB-231 TD cells trap on the steps of the Parsortix cassette and can be imaged with confocal microscopy directly through the transparent Parsortix cassette with sufficient resolution to detect McTNs (Fig. 7a, white arrows). Similarly, MDA-MB-231 TD cells (1 × 10^3^) spiked into a 7.5 ml blood sample could be recovered from the Parsortix cassette and imaged live on TetherChip surfaces (Fig. 7b) allowing positive tumor cell identification with GFP and McTN imaging (Fig. 7b, white arrows) with a fluorescent membrane dye (CellMask-Orange). Sized-based recovery of the spiked MDA-MB-231 TD cells (5 × 10^3^) from 4 ml blood samples was independently conducted using the Vortex Biosciences VTX-1 instrument, which uses microfluidic turbulence at high flow rates to selectively trap larger tumor cells and then release the enriched tumor cells at a lower flow rate [36]. Live MDA-MB-231 TD tumor cells isolated by the VTX-1 were treated with either vehicle control (0.1% DMSO) or Vinorelbine (10 μM) for 1 h and then chemically-fixed to TetherChip surfaces with formaldehyde. High-resolution confocal imaging of the fixed tumor cells was possible through the nanometer-thin tethering surface, and plasma membrane staining with fluorescent WGA revealed Vinorelbine-dependent reduction of McTNs (Fig. 7c, white arrows). McTN analysis of the fixed tethered WGA-stained cells (Fig. 7d) showed Vinorelbine yielded statistically significant changes in both McTN number per cell (Fig. 7e) and average McTN length (Fig. 7f). Considering that the chemical fixation procedure allowed the entire cell to be visualized, the Vinorelbine drug response test had far greater sensitivity than the live cell experiments (Fig. 1) and a 90% power analysis revealed that only 11 cells are necessary to detect differences in McTN tip distance and 17 cells for McTN number, therefore avoiding the need to propagate tumor cells and moving beyond tumor cell growth as the primary indicator of drug response.

## Discussion

In March 2019, *Nature Reviews Clinical Oncology* published a consensus statement by an international working group emphasizing the urgent need to identify therapies that could reduce metastasis and move past the overreliance on the radiological Response Evaluation Criteria for Solid Tumors (RECIST), which depends so heavily on imaging-based measurements of tumor growth, rather than metastatic characteristics [1]. Since the threshold of detection with clinical imaging requires a large foci of at least 10 million tumor cells, the RECIST strategy leads to an inevitable focus on treatments that affect overall tumor size and fails to account for tumor phenotypes or therapy-induced changes that could influence metastatic risk [1, 2]. There is a strong historical parallel between the current clinical limitations and the early failure of transgenic models of breast cancer to identify genetic regulators of metastasis [37]. Since transgenic mice were routinely monitored by macroscopic observation for tumor growth, transgenic lines that produced palpable tumors were maintained while transgenic lines where genetic alterations did not produce a tumor were discarded [37]. As a consequence, transgenic models of breast cancer produced large primary tumors that rarely metastasized and identified almost exclusively genetic determinants of primary tumor growth [37]. Conversely, genetic alterations that could enhance tumor metastasis but did not affect primary tumor growth were largely overlooked [37], until persistent investigators raised awareness of genes that selectively altered metastasis, such as nm23 [38], KISS1 [39] and BRMS1 [40]. The clinical reliance on RECIST measurements of tumor size presents similar barriers today to the discovery of anti-metastasis therapies and effective management of patients to reduce metastatic risk [1]. Emerging evidence also shows that alterations in tumor size can be misleading with respect to metastatic risk and underscore the importance of identifying drug treatments that reduce CTC metastasis [6].

Neoadjuvant chemotherapy treatments of breast tumors in mice can reduce tumor size, but actually yield significant elevations in CTCs that increase metastasis [6]. This treatment-induced CTC shedding and metastasis occurred after chemotherapy treatments which have highly-divergent mechanisms of action, targeting either microtubule stabilization (paclitaxel) or DNA replication (doxorubicin/cyclophosphamide) [6]. Alarmingly, the molecular alterations that accompanied increased CTC shedding and metastasis in mice were also observed in the tumor sections from human breast cancer patients receiving neoadjuvant chemotherapy [6], raising the possibility that chemotherapy could also be increasing metastatic risk in patients. Earlier clinical studies in breast cancer patients demonstrated significant increases in CTCs following neoadjuvant therapy of up to 1000-fold [41]. More recent studies show that Black women with early breast cancer have decreased distant metastasis-free survival with neoadjuvant chemotherapy compared to adjuvant chemotherapy (HR = 3.61, *p* = 0.002), and this difference is not observed in White women (HR = 0.72, *p* = 0.824), revealing disparities in outcome from neoadjuvant chemotherapy that are not yet fully understood [42]. Since CTC viability is not routinely monitored in clinical studies, the different risks of tumors shedding dead CTCs or tumors shedding live CTCs cannot currently be resolved [3]. However, the potential differences in metastatic risk between a tumor shedding dead CTCs or live CTCs seem obvious, and it will be important to eventually distinguish when the treatment-induced changes in radiological tumor size are the result of tumor cell death or tumor cells shedding into the circulation. The GeparTrio trial monitored outcome for 8 years in 2012 hormone-receptor negative breast cancer patients and showed that patients had significantly lower risk of relapse (HR = 0.59, *p* = 0.001) when switched to a Vinorelbine-based regimen after failing to respond radiologically to the combination of docetaxel/doxorubicin/cyclophosphamide [43]. Switching to Vinorelbine as a second-line treatment did not improve overall survival significantly (HR = 0.85, p = 0.432) [43], but given the apparent risks of CTC shedding with both taxanes and doxorubicin/cyclophosphamide [6], it is possible that Vinorelbine would be more effective as a first-line treatment in TNBC. Our results do not establish that this action to reduce metastasis is unique to Vinorelbine, and only provide a proof-of-concept that a focused treatment with Vinorelbine can reduce metastasis more than primary tumor growth. It definitely remains possible that other FDA-approved therapies could have similar anti-metastatic effects, and that will be important to test in future studies.

The limitations of clinical imaging and the risk of CTC shedding also emphasize the need to develop new therapies that reduce CTC metastasis [1, 2]. However, the lack of focus on metastatic determinants also provides the opportunity that existing FDA-approved compounds could have anti-metastatic effects that have been previously unappreciated. In this study, we demonstrated that a targeted 24 h treatment window with the FDA-approved compound Vinorelbine, could yield a significant decrease in CTC metastasis (Fig. 6, Additional file 6: Fig. S6) without significantly improving primary tumor survival (Fig. 5). Moreover, these anti-metastatic benefits were realized with only a single day of treatment and at a human-equivalent dose of Vinorelbine (10.9 mg/m^2^) [34] that is well below the current standard of care dose in breast cancer patients (25–30 mg/m^2^). While there was a trend toward lower initial cell retention in the lungs of Vinorelbine-treated mice, it did not reach statistical significance (Additional file 7: Fig. S7). In both the control and Vinorelbine groups, approximately 90% of the injected tumor cells disappeared from the lungs within 24 h, indicating that even the greater sensitivity of bioluminescence imaging is challenged to detect the small number of retained CTCs above the optical background of host tissue. In contrast, the response of isolated tumor cells to Vinorelbine could be measured using a computational McTN analysis in only one hour without requiring long-term tumor cell growth. Current models to analyze patient tumor cells, such as spheroids and patient-derived xenografts (PDX) require tumor cell propagation that can impose confounding adaptive and selective pressures on the tumor cells that eventually grow in these systems [44].

More importantly, up to 50% of breast cancer PDX fail to grow in mice, leading to a significant loss of patient representation [44]. As one example of measuring metastatic phenotype without requiring growth, we recently reported that a microfluidic tumor cell migration assay can characterize metastatic potential within 13 h [18].

The McTN analysis technique reported here provides a new approach to rapidly assess tumor cell drug response against the metastatic phenotype of McTNs within 1 h, and further reduces the complications and variables that are introduced by requiring long-term tumor cell propagation. The ability of Vinorelbine to reduce the metastatic phenotypes of tumor cell reattachment and clustering could also be measured within 4 h (Figs. 3, 4, Additional file 4: Fig. S4, Additional file 5: Fig. S5). It is important to recognize that McTNs in this study are used primarily as a rapid indicator of drug response, and future studies will be needed to definitively prove that it is drug action against McTNs that directly suppress metastasis. Since McTNs are also an indicator of tumor cell mechanical properties [45], it remains possible that suppression of McTNs by Vinorelbine reflects a change in tumor cell mechanics that makes CTCs more likely to fragment in the bloodstream, rather than the ability for Vinorelbine to directly reduce McTN-dependent blood vessel wall attachment. To move toward a clinical assay for McTNs, it has also been necessary to develop technologies that help improve McTN imaging and preservation. Previous electron microscopy studies have shown that McTNs are only ~ 100 nm wide and highly sensitive to fixation conditions [46]. In this study, we optimized TetherChip microfluidic cell tethering technology [20, 21] to allow a rapid chemical fixation procedure that preserves McTNs on tumor cells, even after isolation from blood samples (Fig. 7c, d). The small number of CTCs recovered from breast cancer patient blood samples has proven challenging for outgrowth as either PDX or spheroids [44], but the comparatively small number of cells required for this McTN analysis (as few as 11 cells for McTN length) could eventually be more achievable for liquid biopsy samples [3]. Incorporating new microfluidic technologies that enable capture of live CTCs, like the ANGLE Parsortix [35] and the Vortex VTX-1 [47], now demonstrate that it is possible to conduct McTN analysis on live tumor cells isolated from blood samples and rapidly detect the reduction in McTNs caused by Vinorelbine (Fig. 7e, f).

Cancer drugs that broadly disrupt microtubules have long proven effective at reducing tumor growth and improving patient survival, but also have significant toxicities that limit dosing [19]. As the understanding of tumor cell phenotypes that promote metastasis is clarified [1, 3], it may be possible to further refine the treatment window or target specific subsets of microtubules to reduce toxicities to normal tissues. The focused 24 h treatment with Vinorelbine that we report here reduced McTNs and metastasis at well below the maximum-tolerated dose. It is also possible to decrease McTNs by selectively targeting tubulin detyrosination [24] and acetylation [12], which could help minimize side effects of broad microtubule disruption with existing FDA-approved cancer drugs [19]. We have previously shown that reducing microtubule detyrosination with either Parthenolide or Curcumin can reduce McTNs and tumor cell reattachment, without the overall microtubule network disruption that is caused by current microtubule-disrupting cancer drugs [24, 48]. The current study establishes a proof-of-concept that it is possible to use a focused treatment of FDA-approved Vinorelbine to reduce CTC metastasis, but it will also remain important to determine the ideal window for such an anti-metastasis treatment strategy. Given that CTCs and metastasis can be increased by multiple types of primary tumor treatment (surgery [49], radiation [50], chemotherapy [6], photodynamic therapy [51]), the most likely opportunity for therapies to reduce CTC metastasis would be combination therapy at the time of primary tumor treatment, helping to ensure that CTCs shed by that primary tumor treatment have reduced metastatic potential. Our current data indicate that Vinorelbine could serve as one potential combination therapy to reduce CTC metastasis, but also indicate that preclinical and clinical studies which rely primarily on tumor growth could overlook such anti-metastatic actions of existing FDA-approved therapies.

## Supplementary Information

**Additional file 1: Fig. S1**. Vinorelbine decreases McTNs and perimeter on breast tumor cells. MDA-MB-231 TD, MDA-MB-436 TD and BT-549 TD cells were tethered onto a TetherChip surface and stained with the cell membrane dye WGA (1:100) and nuclear dye Hoechst 33,258 (1:5000). **A**) Representative confocal images taken at 60 × magnification using an Olympus IX81 microscope with a Fluoview FV1000 confocal laser scanning system. Scale bars correspond to 10 μm. **B**) Vinorelbine treatment (10 μM) for 1 h. results in a significant decrease in McTN frequency (%) compared to vehicle control. McTN scoring consists of mean values from four independent experiments where 100 cells were blindly counted and averaged. **C**) Twenty cells imaged from panel (A) were analyzed for perimeter quantification using Fiji ImageJ. Compared to the vehicle control treated MDA-MB-231 TD, MDA-MB-436 TD and BT-549 TD cells, Vinorelbine significantly reduced the perimeter of cells. A reduction in perimeter of a cell correlates with less McTNs. **D**) Representative raw images of tethered cells and computer determined cell body outline (blue), cell perimeter (red), and McTNs tips (yellow). **E**) Live cell analysis measuring an average number of McTN tips for cells treated with vehicle and Vinorelbine (10 μM, 1 h.). McTN number is the number of McTNs per cell. McTN distance is average distance of McTN tips from cell body boundary. For live cell image analysis, a population of 25 cells per condition was analyzed from 3 independent experimental replicate.

**Additional file 2: Fig. S2**. Cell viability after Vinorelbine treatment **A**) MDA-MB-231 TD cells treated with a dose range of Vinorelbine for 24 h (black) and 48 h (gray) shows a dose-dependent decrease in cell viability over time. Staurosporine (1 µM) was used as a positive control to promote cell death and decrease cell viability. Vinorelbine (10 μM) caused minimal toxicity after 24 h (black arrow). Data are shown as mean ± SD, n = 3. **B**) Representative brightfield images were taken for each condition using the Nikon Eclipse Ti2-E inverted microscope at 10 × magnification. MDA-MB-231 TD cells treated with Vinorelbine (10 µM) or Staurosporine (1 µM) for 24 h and 48 h. Scale bar = 100 µm. **C**) Cell viability assay with Vinorelbine (10 µM, 24 h and 48 h) in MDA-MB-231 TD, MDA-MB-436 TD and BT-549 TD cell lines. Vinorelbine treatment for 24 h (black) and 48 h (gray) shows a similar dose-dependent response as shown in panel A. Staurosporine (1 µM) shown in hatched bars, was compared to the Vinorelbine treatment and used a positive control for 24 h and 48 h. **D**) Representative phase contrast images of vehicle control and Vinorelbine-treated MDA-MB-436 TD and BT-549 TD cells over time to visualize cell attachment or lack of attachment (rounding). Panels taken at a 10 × magnification. Scale bar = 100 µm.

**Additional file 3: Fig. S3**. Vinorelbine decreases the filamentous microtubule network. **A**) Immunoblot analysis of Vinorelbine (10 μM) treated MDA-MB-436 TD and BT-549 TD for 24 h and 48 h. The cytoskeletal post-translational modification acetylated tubulin (acetyl-tubulin) and total α-tubulin remain unchanged at 24 h and 48 h, compared with vehicle control (0.1% DMSO). Vinorelbine treatment in both the MDA-MB-436 TD and BT-549 TD decreases detyrosinated tubulin (Glu-tubulin) with time. **B**) Representative immunofluorescence images of tethered and fixed MDA-MB-436 TD and BT-549 TD after 1 h treatment of Vinorelbine (10 µM). The filamentous tubulin structures (acetyl-tubulin, and glu-tubulin) are destroyed (green), while the nuclear stain Hoechst (blue) remains intact. Cells were stained with Hoechst 33,258 (1:5000, blue), α-tubulin, acetyl-tubulin, and glu-tubulin (1:1000, green) and were taken at 40 × magnification using an Olympus IX81 microscope with a Fluoview FV1000 confocal laser scanning system. Scale bar = 10 µm.

**Additional file 4: Fig. S4**. Vinorelbine treatment decreases tumor cell reattachment. **A**) Reattachment efficiency of the MDA-MB-436 TD and BT-549 TD cells treated with Vinorelbine (10 μM) is significantly lower than vehicle control treated cells (0.1% DMSO). Changes in impedance are apparent as early as 4 h and significant differences continue for 24 h after initial seeding. Representative experiment from three independent experiments; each performed in quadruplicate. **B**) Representative brightfield images of MDA-MB-436 TD and Bt-549 cells. Images were taken at 10 × magnification at 0, 24, and 48 h in vehicle control, (0.1% DMSO). Vinorelbine (10 μM), and Staurosporine(1 µM). Scale bar = 100 µm.

**Additional file 5: Fig. S5**. Vinorelbine treatment decreases homotypic cluster aggregation in vitro. Representative Hoechst stained images of the full ROI region of the ibidi slide. Images of control and Vinorelbine-treated MDA-MB-231 TD, MDA-MB-436 TD and BT-549 TD cells imaged over time to visualize cluster formation efficiency. Images taken at 4 × magnification.

**Additional file 6: Fig. S6**. Lung retention and metastatic development in the presence of Vinorelbine. NSG/SCID mice treated with vehicle control (0.1% DMSO) or 5 mg/kg Vinorelbine during a single 24 h dose prior to injection with either the MDA-MB-436 TD or BT-549 TD cell lines. Animals were treated once at 24 h before cell inoculation due to the severe immune deficiency of the host animal. **A**) Representative bioluminescence images of mice treated and injected with MDA-MB-436 TD cells. In the MDA-MB-436 TD line, the vehicle control population resulted in 5 out of 5 animals (100%) exhibiting tumor formation in lung tissue at 7 weeks post inoculation with 4/5 (80%) surviving. The surviving vehicle control animals all have at least a tenfold increase over the initial bioluminescence value. The Vinorelbine treatment resulted in 8 out of 8 animals (100%) with disease-free survival at 7 weeks. The surviving Vinorelbine treated animals did not meet or exceed their initial value. **B**) Fold differences of retained bioluminescence in the lung of animals inoculated MDA-MB-436 TD cells via tail vein. **C**) Representative bioluminescence images of mice treated and injected with BT-549 TD cells. The BT-549 TD cells treated with the vehicle control resulted in 4 out of 5 animals (80%) exhibiting tumor formation in lung tissue at 7 weeks post inoculation with 5/5 (100%) surviving. 4/5 (80%) of the surviving vehicle control animals have a minimum of a twofold increase over the initial bioluminescence value. The Vinorelbine treatment resulted in 7 out of 8 animals (87.5%) with disease-free survival at 7 weeks. The surviving Vinorelbine treated animals did not meet or exceed their initial value. Photon flux color scale is shown to the right. Data represent individual animal examined and measured as a fold change of the initial value of each independent animal.

**Additional file 7: Fig. S7**. Vinorelbine treatment does not significantly reduce early lung retention. **A.** Representative bioluminescence images of mice after introducing the MDA-MB-231 TD cells via tail vein. Mice received 2 injections (24 h and 2 h) of DMSO control (0.1%) or Vinorelbine (5 mg/kg) prior to cell inoculation and imaging. At all timepoints, there is slightly lower lung bioluminescence in Vinorelbine-treated mice, but the average differences (**B**) do not reach statistical significance (P = 0.556).

## Data Availability

All data generated or analyzed during this study are included in this published article [and its supplementary information files.

## Abbreviations

McTN: Microtentacle
CTC: Circulating tumor cell
TNBC: Triple negative breast cancer
NCCN: National comprehensive cancer network
ATCC: American type culture collection
PMA: Poly(methacrylic) acid
PAAm: Polyacrylamide
PEM: Polyelectrolyte multilayers
DOTAP: 1,2-Dioleoyl-3-trimethylammonium-propane
WGA: Wheat germ agglutinin
DMSO: Dimethyl sulfoxide
RECIST: Response evaluation criteria for solid tumors
PDX: Patient-derived xenografts
