# Supplementary figures and images for: Microtubule disruption reduces metastasis more effectively than primary tumor growth
Source: Breast Cancer Res. 2022 Feb 14;24:13. doi: 10.1186/s13058-022-01506-2 (PMC8842877; doi:10.1186/s13058-022-01506-2)

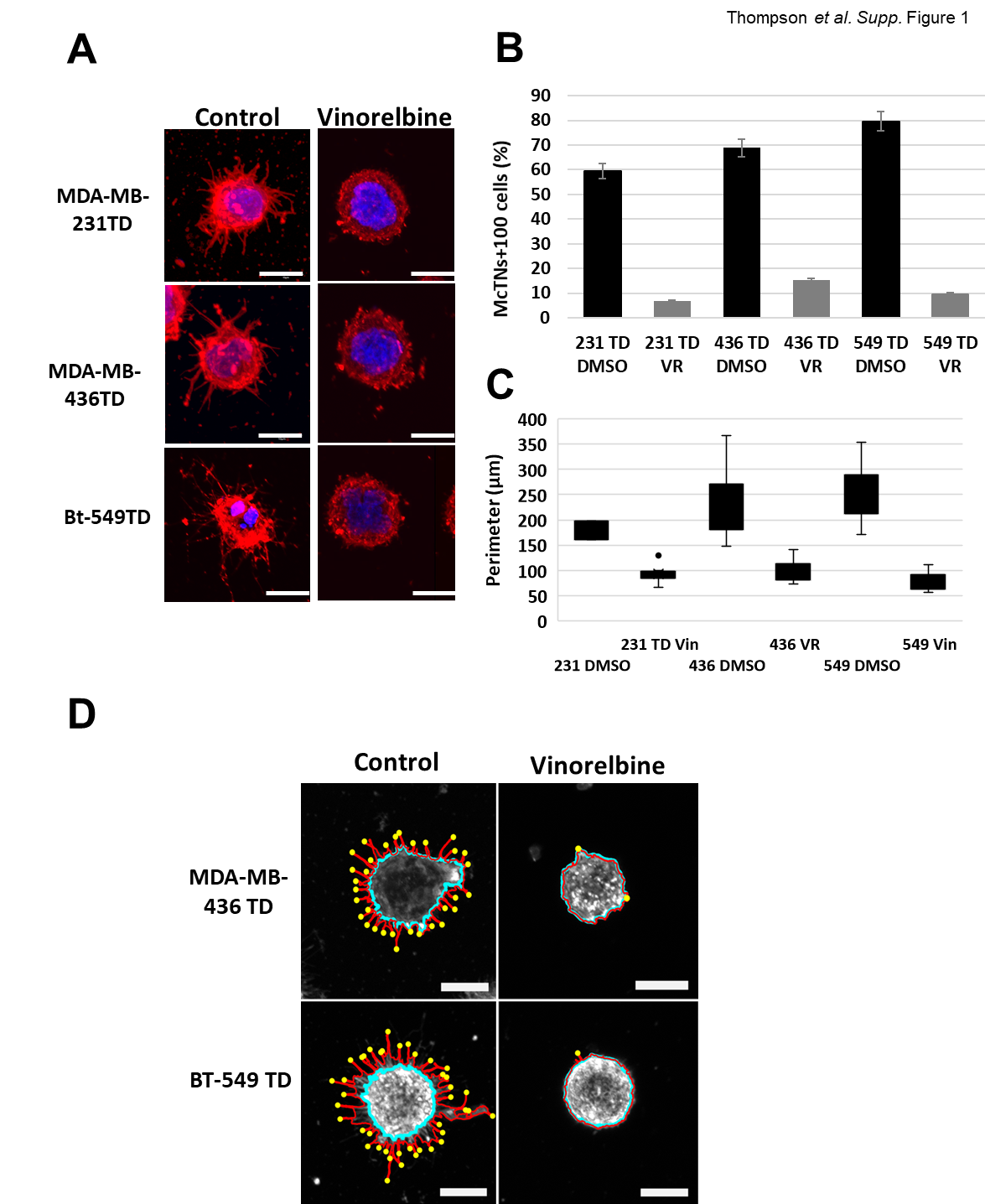


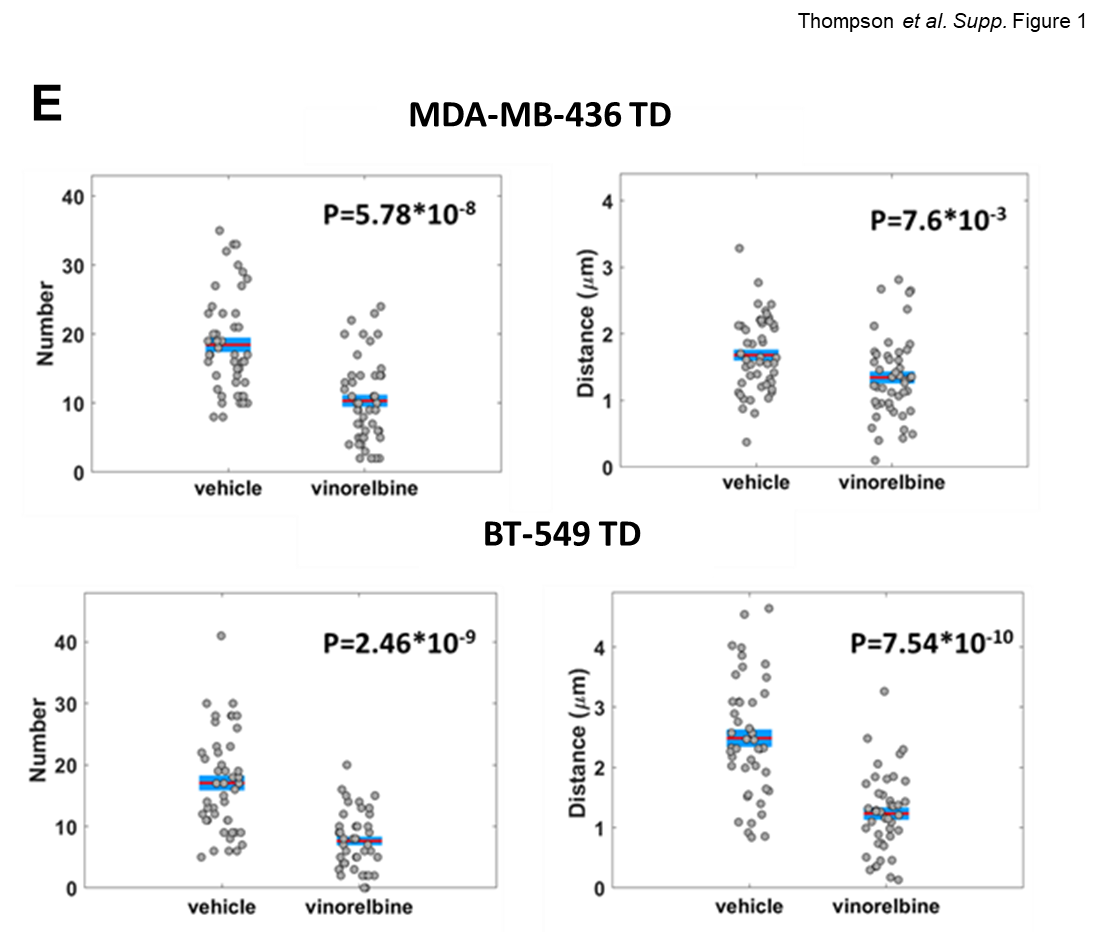

Supplement: Supplementary file 1 — Additional file 1: Fig. S1. Vinorelbine decreases McTNs and perimeter on breast tumor cells. MDA-MB-231 TD, MDA-MB-436 TD and BT-549 TD cells were tethered onto a TetherChip surface and stained with the cell membrane dye WGA (1:100) and nuclear dye Hoechst 33,258 (1:5000). A) Representative confocal images taken at 60 × magnification using an Olympus IX81 microscope with a Fluoview FV1000 confocal laser scanning system. Scale bars correspond to 10 μm. B) Vinorelbine treatment (10 μM) for 1 h. results in a significant decrease in McTN frequency (%) compared to vehicle control. McTN scoring consists of mean values from four independent experiments where 100 cells were blindly counted and averaged. C) Twenty cells imaged from panel (A) were analyzed for perimeter quantification using Fiji ImageJ. Compared to the vehicle control treated MDA-MB-231 TD, MDA-MB-436 TD and BT-549 TD cells, Vinorelbine significantly reduced the perimeter of cells. A reduction in perimeter of a cell correlates with less McTNs. D) Representative raw images of tethered cells and computer determined cell body outline (blue), cell perimeter (red), and McTNs tips (yellow). E) Live cell analysis measuring an average number of McTN tips for cells treated with vehicle and Vinorelbine (10 μM, 1 h.). McTN number is the number of McTNs per cell. McTN distance is average distance of McTN tips from cell body boundary. For live cell image analysis, a population of 25 cells per condition was analyzed from 3 independent experimental replicate. [file 13058_2022_1506_MOESM1_ESM.docx]

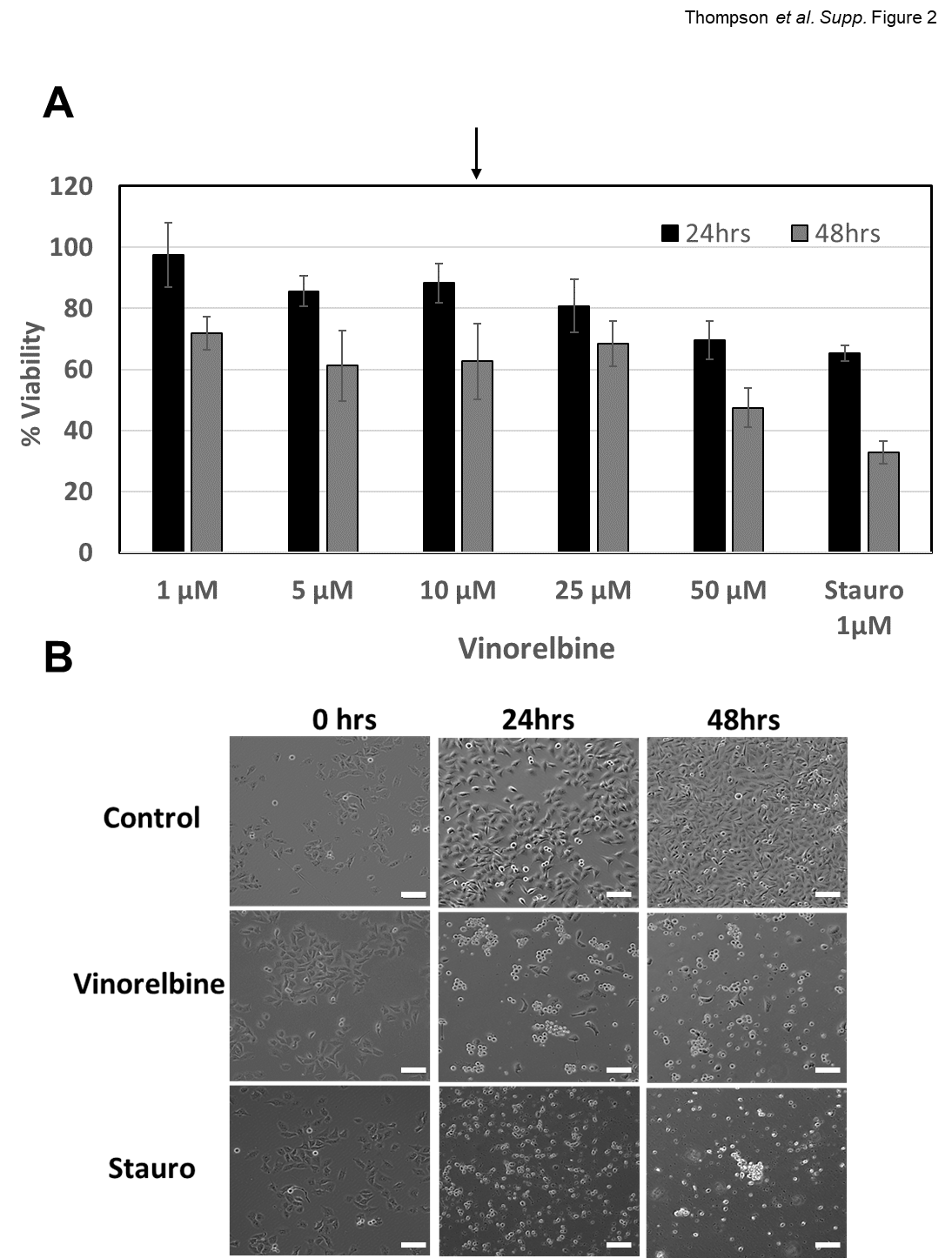


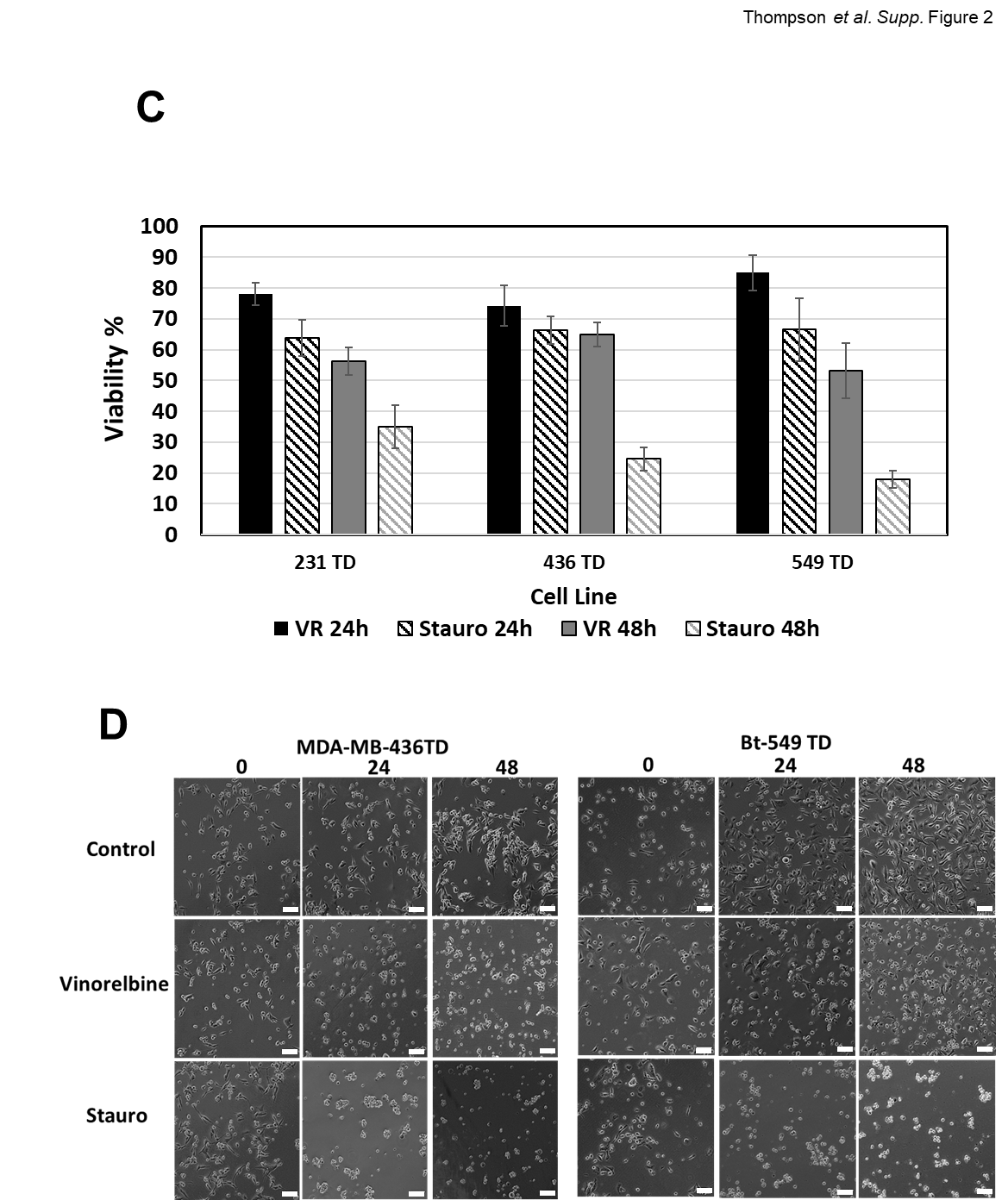

Supplement: Supplementary file 2 — Additional file 2: Fig. S2. Cell viability after Vinorelbine treatment A) MDA-MB-231 TD cells treated with a dose range of Vinorelbine for 24 h (black) and 48 h (gray) shows a dose-dependent decrease in cell viability over time. Staurosporine (1 µM) was used as a positive control to promote cell death and decrease cell viability. Vinorelbine (10 μM) caused minimal toxicity after 24 h (black arrow). Data are shown as mean ± SD, n = 3. B) Representative brightfield images were taken for each condition using the Nikon Eclipse Ti2-E inverted microscope at 10 × magnification. MDA-MB-231 TD cells treated with Vinorelbine (10 µM) or Staurosporine (1 µM) for 24 h and 48 h. Scale bar = 100 µm. C) Cell viability assay with Vinorelbine (10 µM, 24 h and 48 h) in MDA-MB-231 TD, MDA-MB-436 TD and BT-549 TD cell lines. Vinorelbine treatment for 24 h (black) and 48 h (gray) shows a similar dose-dependent response as shown in panel A. Staurosporine (1 µM) shown in hatched bars, was compared to the Vinorelbine treatment and used a positive control for 24 h and 48 h. D) Representative phase contrast images of vehicle control and Vinorelbine-treated MDA-MB-436 TD and BT-549 TD cells over time to visualize cell attachment or lack of attachment (rounding). Panels taken at a 10 × magnification. Scale bar = 100 µm. [file 13058_2022_1506_MOESM2_ESM.docx]

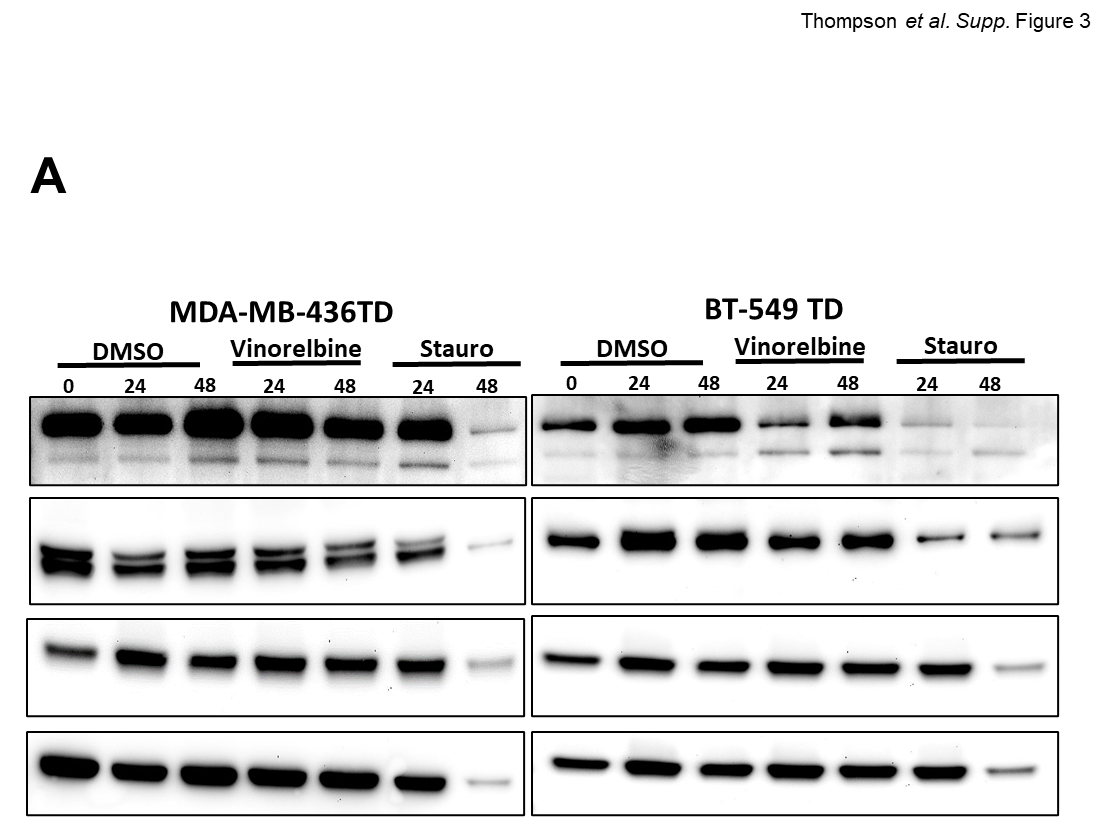


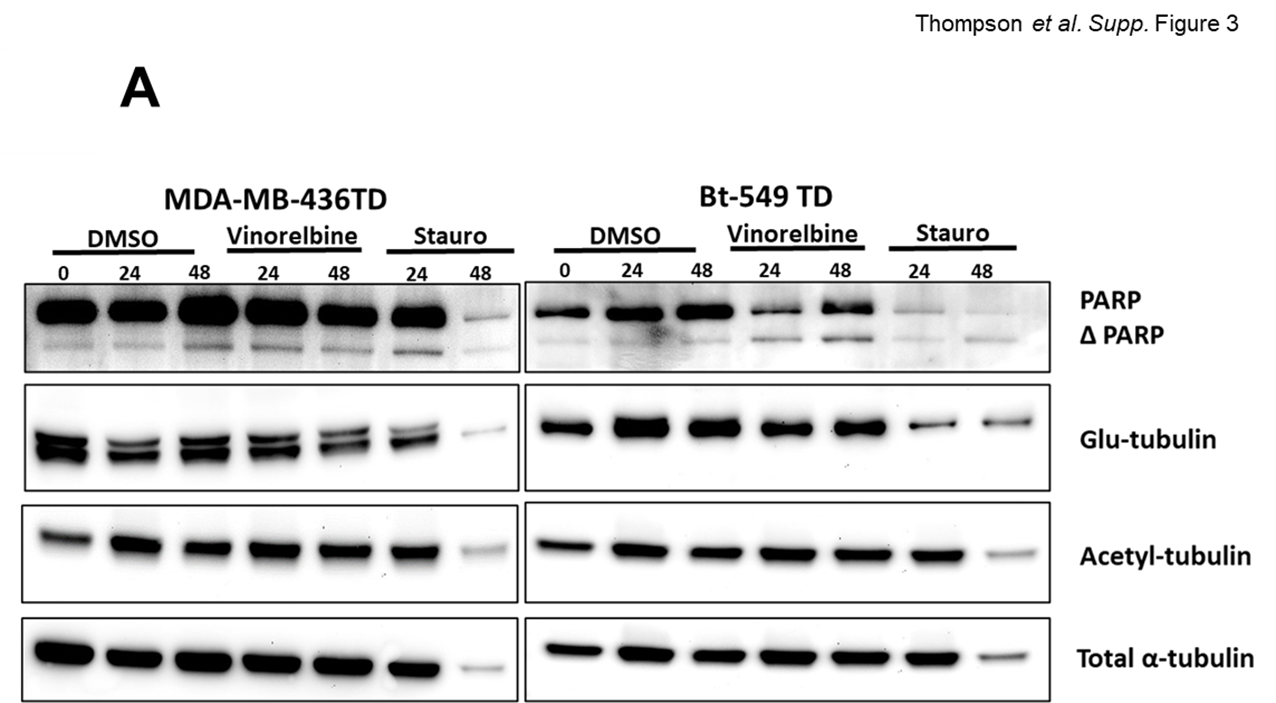


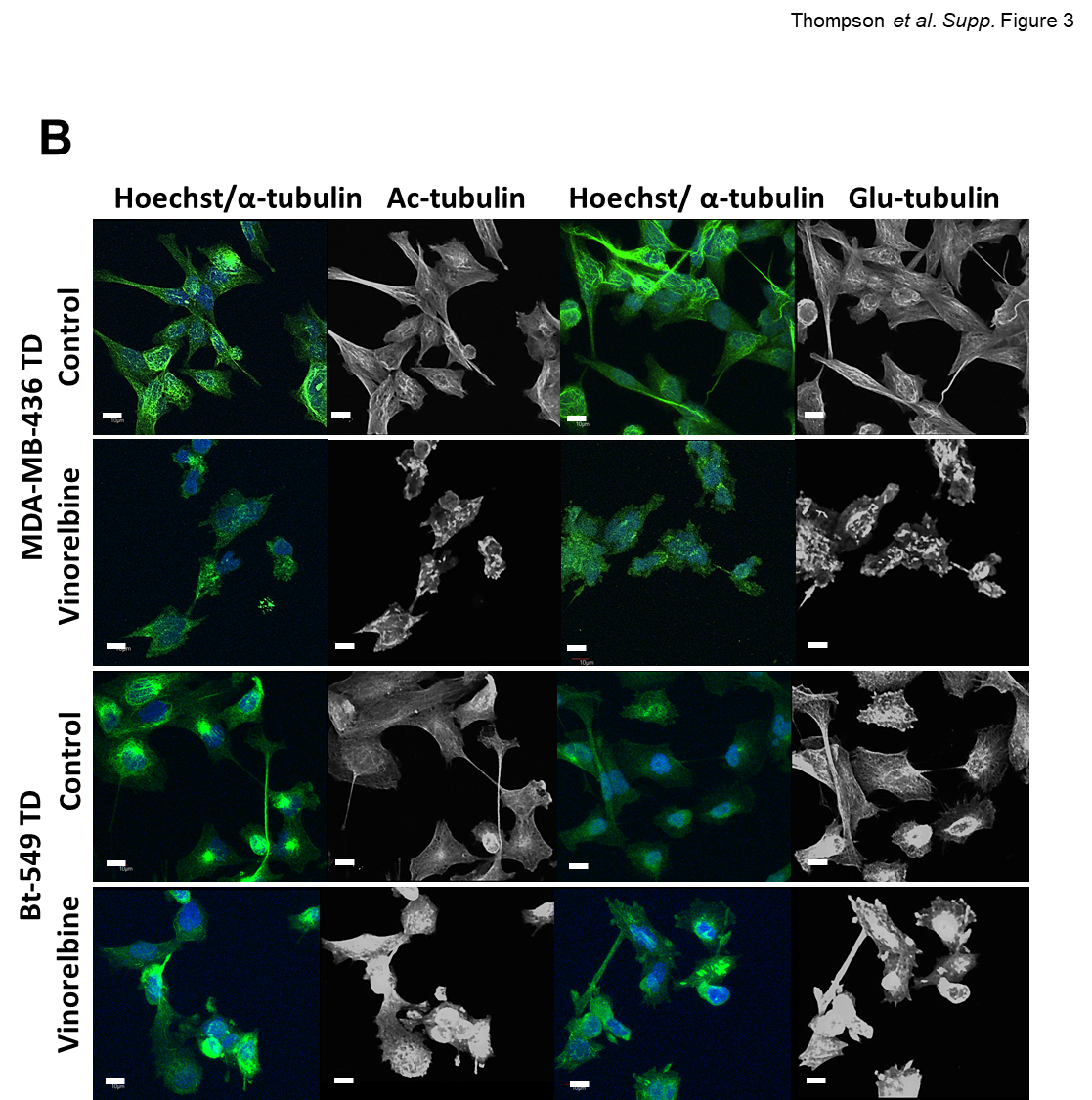

Supplement: Supplementary file 3 — Additional file 3: Fig. S3. Vinorelbine decreases the filamentous microtubule network. A) Immunoblot analysis of Vinorelbine (10 μM) treated MDA-MB-436 TD and BT-549 TD for 24 h and 48 h. The cytoskeletal post-translational modification acetylated tubulin (acetyl-tubulin) and total α-tubulin remain unchanged at 24 h and 48 h, compared with vehicle control (0.1% DMSO). Vinorelbine treatment in both the MDA-MB-436 TD and BT-549 TD decreases detyrosinated tubulin (Glu-tubulin) with time. B) Representative immunofluorescence images of tethered and fixed MDA-MB-436 TD and BT-549 TD after 1 h treatment of Vinorelbine (10 µM). The filamentous tubulin structures (acetyl-tubulin, and glu-tubulin) are destroyed (green), while the nuclear stain Hoechst (blue) remains intact. Cells were stained with Hoechst 33,258 (1:5000, blue), α-tubulin, acetyl-tubulin, and glu-tubulin (1:1000, green) and were taken at 40 × magnification using an Olympus IX81 microscope with a Fluoview FV1000 confocal laser scanning system. Scale bar = 10 µm. [file 13058_2022_1506_MOESM3_ESM.docx]

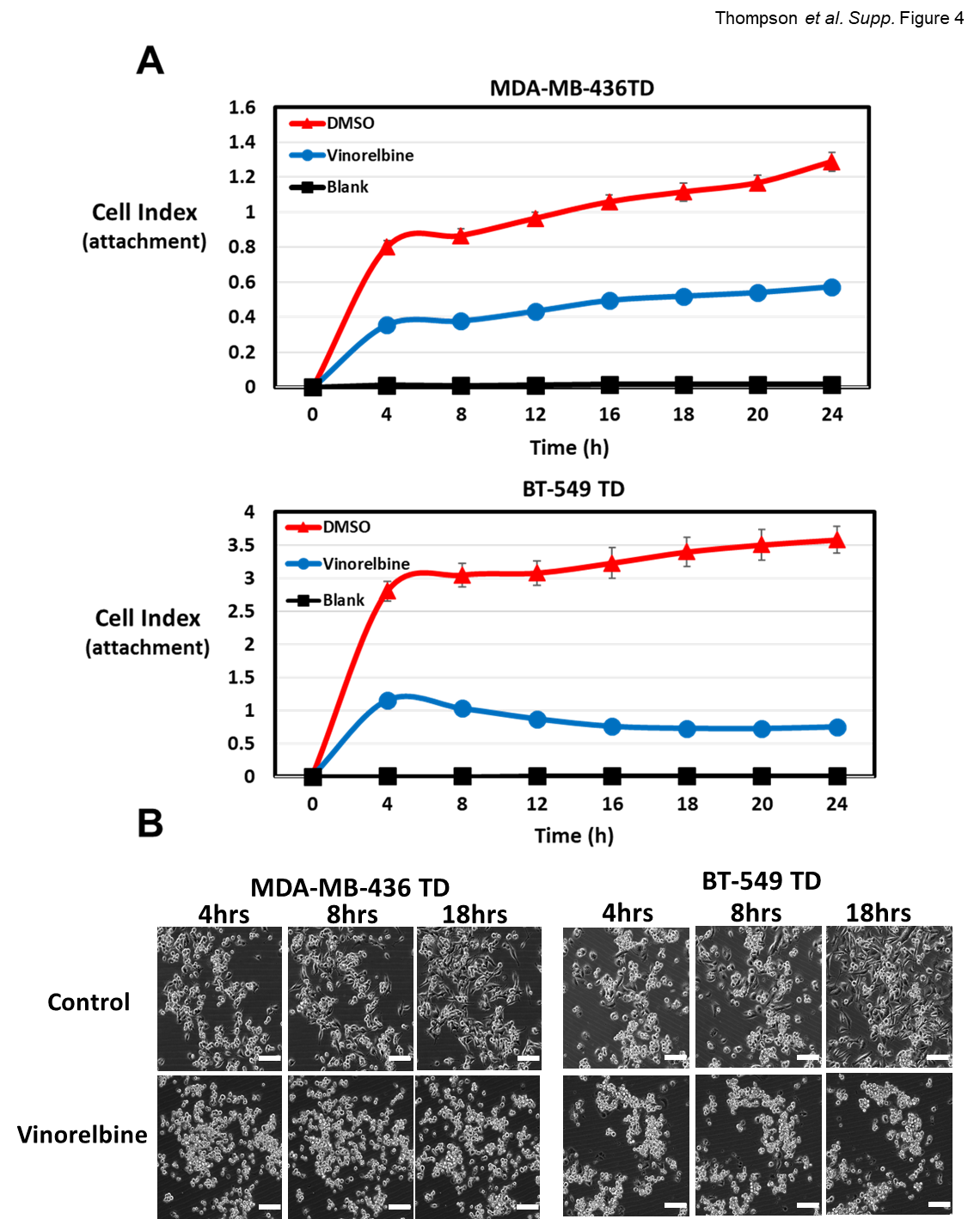

Supplement: Supplementary file 4 — Additional file 4: Fig. S4. Vinorelbine treatment decreases tumor cell reattachment. A) Reattachment efficiency of the MDA-MB-436 TD and BT-549 TD cells treated with Vinorelbine (10 μM) is significantly lower than vehicle control treated cells (0.1% DMSO). Changes in impedance are apparent as early as 4 h and significant differences continue for 24 h after initial seeding. Representative experiment from three independent experiments; each performed in quadruplicate. B) Representative brightfield images of MDA-MB-436 TD and Bt-549 cells. Images were taken at 10 × magnification at 0, 24, and 48 h in vehicle control, (0.1% DMSO). Vinorelbine (10 μM), and Staurosporine(1 µM). Scale bar = 100 µm. [file 13058_2022_1506_MOESM4_ESM.docx]

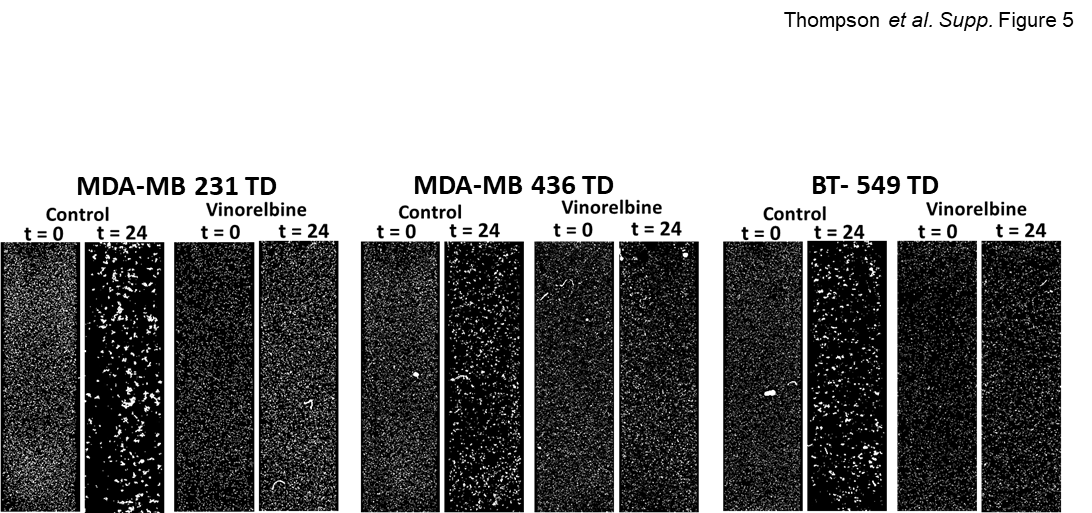

Supplement: Supplementary file 5 — Additional file 5: Fig. S5. Vinorelbine treatment decreases homotypic cluster aggregation in vitro. Representative Hoechst stained images of the full ROI region of the ibidi slide. Images of control and Vinorelbine-treated MDA-MB-231 TD, MDA-MB-436 TD and BT-549 TD cells imaged over time to visualize cluster formation efficiency. Images taken at 4 × magnification. [file 13058_2022_1506_MOESM5_ESM.docx]

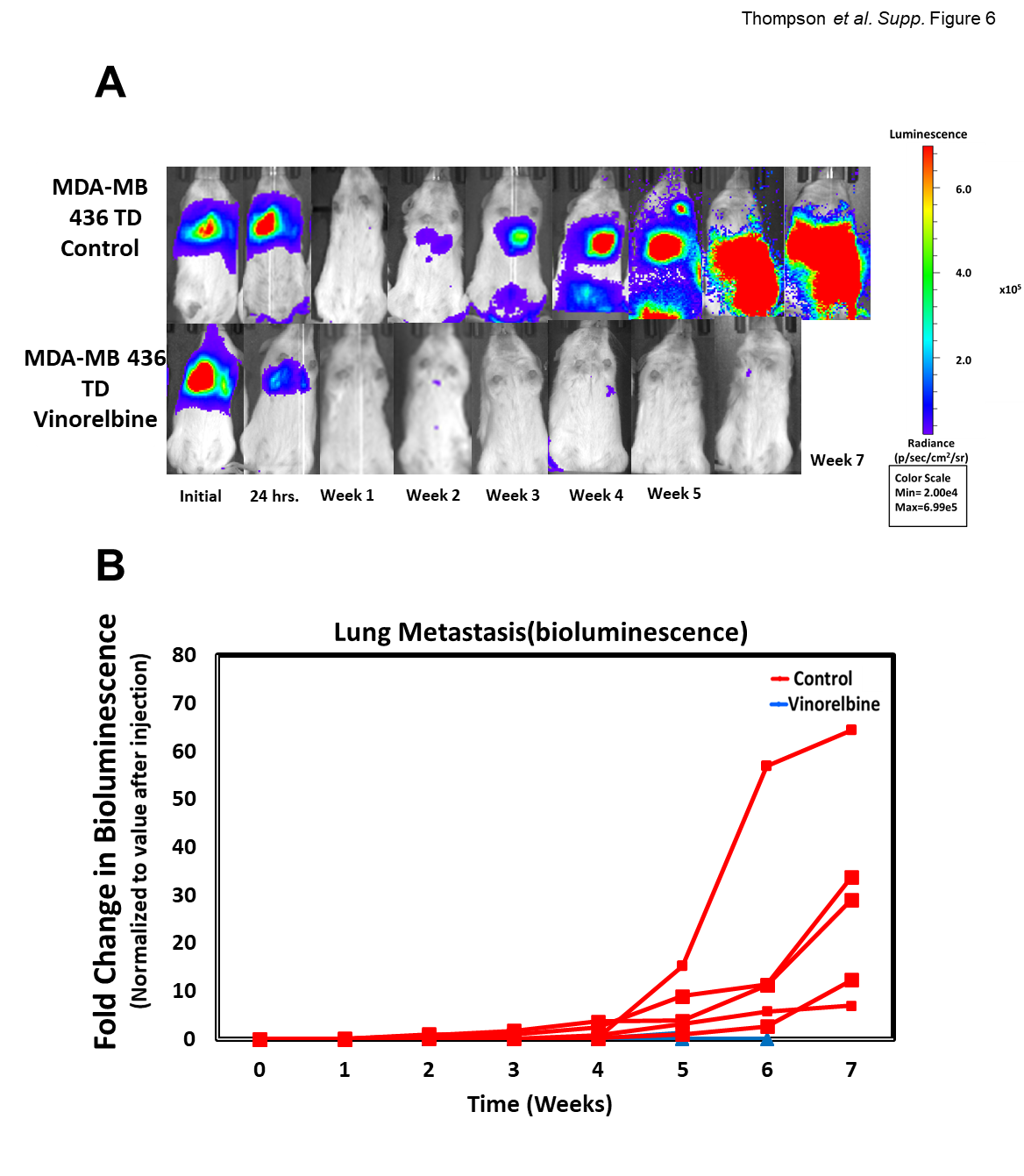


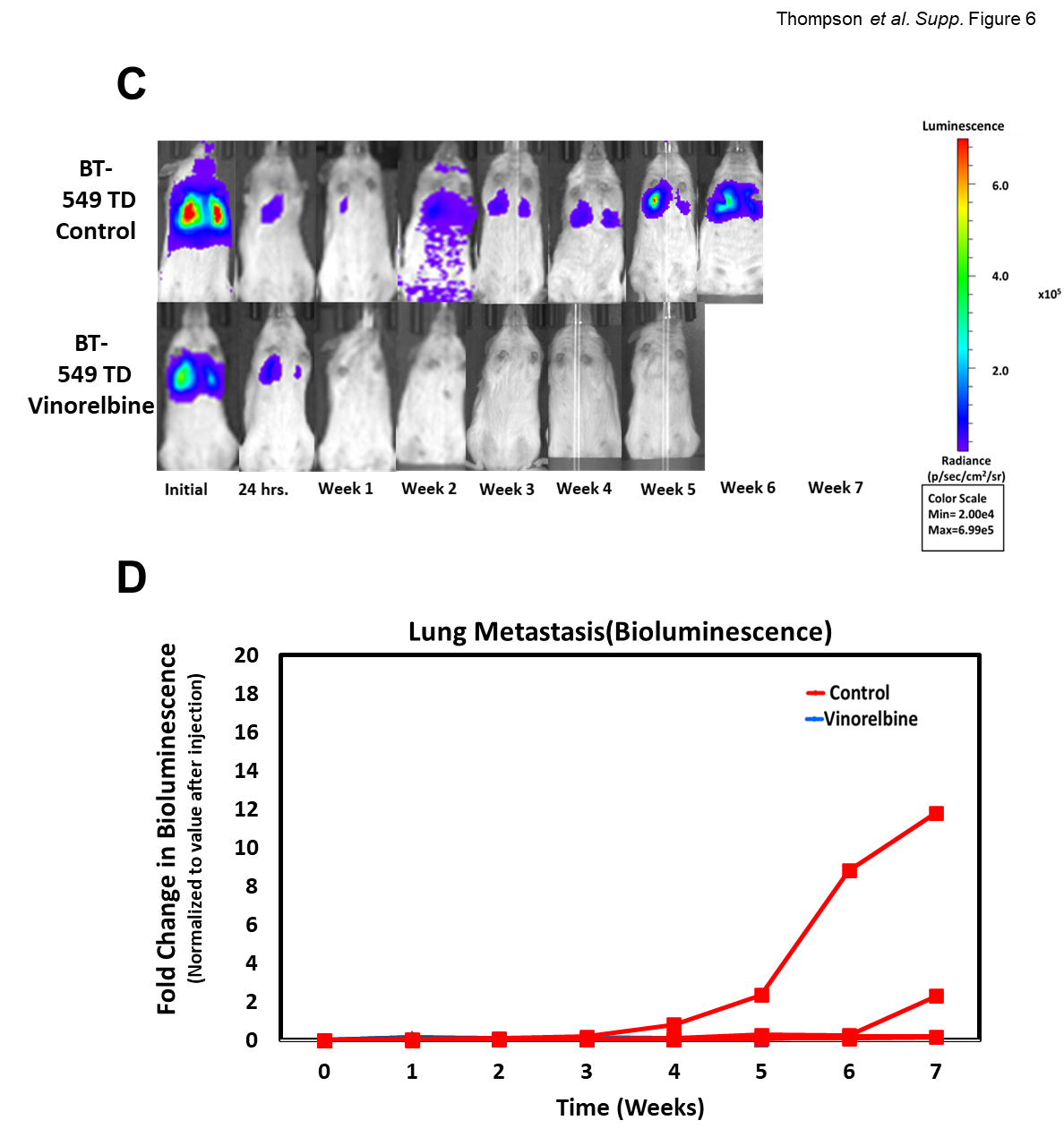

Supplement: Supplementary file 6 — Additional file 6: Fig. S6. Lung retention and metastatic development in the presence of Vinorelbine. NSG/SCID mice treated with vehicle control (0.1% DMSO) or 5 mg/kg Vinorelbine during a single 24 h dose prior to injection with either the MDA-MB-436 TD or BT-549 TD cell lines. Animals were treated once at 24 h before cell inoculation due to the severe immune deficiency of the host animal. A) Representative bioluminescence images of mice treated and injected with MDA-MB-436 TD cells. In the MDA-MB-436 TD line, the vehicle control population resulted in 5 out of 5 animals (100%) exhibiting tumor formation in lung tissue at 7 weeks post inoculation with 4/5 (80%) surviving. The surviving vehicle control animals all have at least a tenfold increase over the initial bioluminescence value. The Vinorelbine treatment resulted in 8 out of 8 animals (100%) with disease-free survival at 7 weeks. The surviving Vinorelbine treated animals did not meet or exceed their initial value. B) Fold differences of retained bioluminescence in the lung of animals inoculated MDA-MB-436 TD cells via tail vein. C) Representative bioluminescence images of mice treated and injected with BT-549 TD cells. The BT-549 TD cells treated with the vehicle control resulted in 4 out of 5 animals (80%) exhibiting tumor formation in lung tissue at 7 weeks post inoculation with 5/5 (100%) surviving. 4/5 (80%) of the surviving vehicle control animals have a minimum of a twofold increase over the initial bioluminescence value. The Vinorelbine treatment resulted in 7 out of 8 animals (87.5%) with disease-free survival at 7 weeks. The surviving Vinorelbine treated animals did not meet or exceed their initial value. Photon flux color scale is shown to the right. Data represent individual animal examined and measured as a fold change of the initial value of each independent animal. [file 13058_2022_1506_MOESM6_ESM.docx]

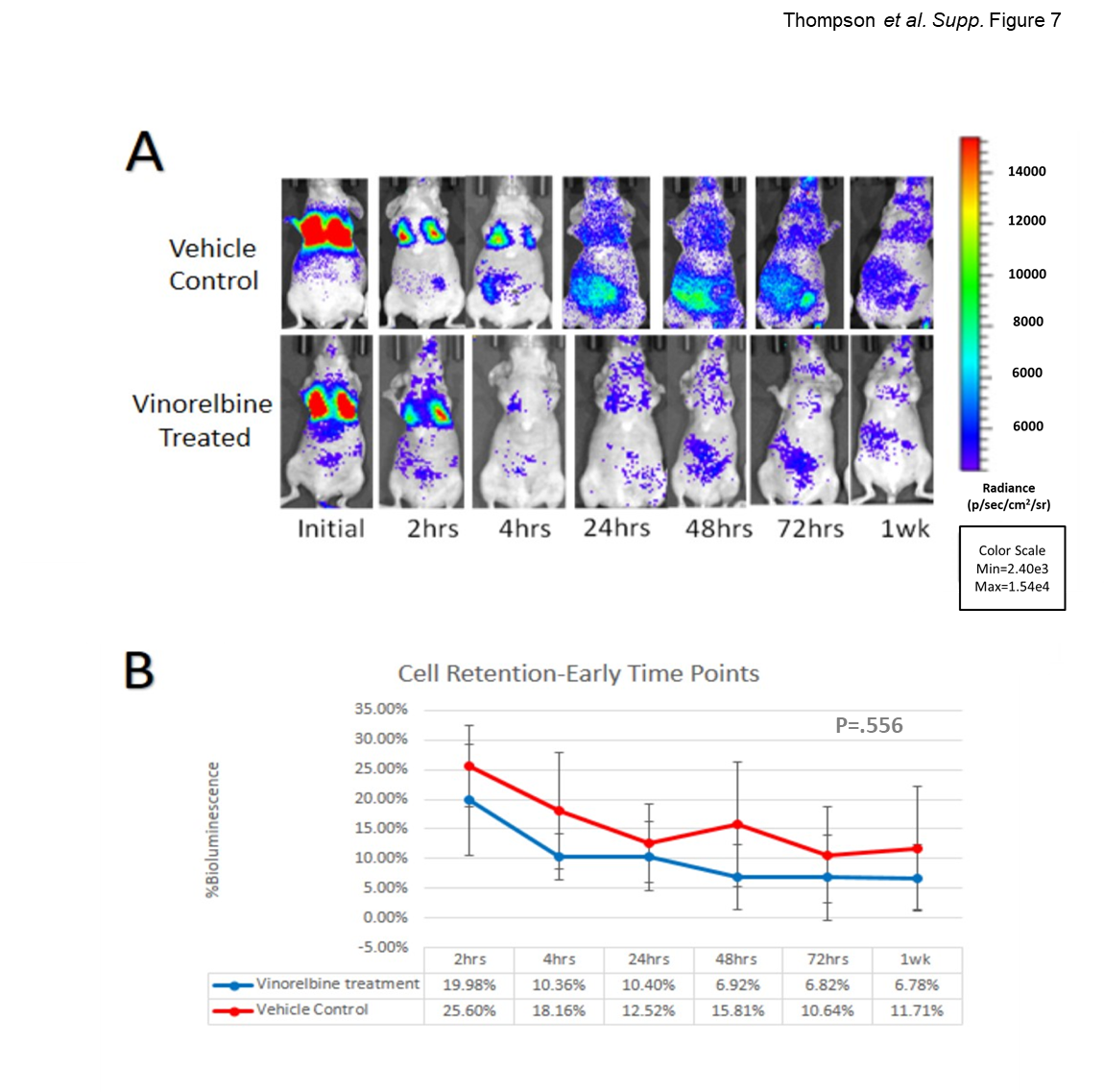

Supplement: Supplementary file 7 — Additional file 7: Fig. S7. Vinorelbine treatment does not significantly reduce early lung retention. A. Representative bioluminescence images of mice after introducing the MDA-MB-231 TD cells via tail vein. Mice received 2 injections (24 h and 2 h) of DMSO control (0.1%) or Vinorelbine (5 mg/kg) prior to cell inoculation and imaging. At all timepoints, there is slightly lower lung bioluminescence in Vinorelbine-treated mice, but the average differences (B) do not reach statistical significance (P = 0.556). [file 13058_2022_1506_MOESM7_ESM.docx]
